# Effect modification by chronic obstructive pulmonary disease, anion gap, and serum creatinine on the association between invasive mechanical ventilation and 28-day mortality in intensive care unit sepsis patients: a retrospective cohort study

**DOI:** 10.3389/fmed.2026.1758229

**Published:** 2026-05-27

**Authors:** Yan Xue, Shao-Xiong Chen, Guo-Ren Chen, Wei Lin, Xing-Feng Yu, Hao Lin, Ran Chen

**Affiliations:** 1 Department of Clinical Pharmacy, Fuqing City Hospital Affiliated to Fujian Medical University, Fuzhou, Fujian, China; 2 Department of Critical Care Medicine, Fuqing City Hospital Affiliated to Fujian Medical University, Fuzhou, Fujian, China; 3 Department of General Surgery, Fuqing City Hospital Affiliated to Fujian Medical University, Fuzhou, Fujian, China

**Keywords:** anion gap, chronic obstructive pulmonary disease, comorbidities, intensive care unit, mechanical ventilation, mortality, sepsis, serum creatinine

## Abstract

**Background:**

Sepsis remains a leading cause of mortality in intensive care units (ICUs) worldwide, affecting millions of patients annually. Although mechanical ventilation (MV) is a life-saving intervention in sepsis management, its association with patient outcomes remains controversial, with conflicting evidence regarding its impact on mortality. Current literature lacks a comprehensive analysis of the clinical phenotypes and laboratory parameters that may modify this relationship. This study aimed to evaluate the association between MV and 28-day mortality in ICU sepsis patients and to explore potential effect modifiers.

**Methods:**

We conducted a retrospective cohort study of adult sepsis patients admitted to the ICU of Fuqing Affiliated Hospital, Fujian Medical University, between January 1, 2017, and December 31, 2024. Sepsis was defined according to Sepsis-3 criteria. The primary exposure was invasive mechanical ventilation, and the primary outcome was 28-day all-cause mortality. Covariates included 25 variables encompassing demographics, seven comorbidities, Acute Physiology and Chronic Health Evaluation II (APACHE II) score, vital signs, eleven laboratory parameters, and antibacterial therapy. We performed multi-variable logistic regression adjusting for these covariates to assess the MV–mortality association, with stratified analyses and interaction terms to evaluate effect modification.

**Results:**

Among 673 ICU sepsis patients, 507 (75.3%) received mechanical ventilation. The 28-day mortality rate was significantly higher in the MV group than in the non-ventilation group (56.0% vs. 29.5%, *p* < 0.001). Multi-variable analysis demonstrated that MV was independently associated with increased 28-day mortality (adjusted odds ratio [OR] 2.62, 95% CI 1.67–4.11, *p* < 0.001). Stratified analyses revealed that chronic obstructive pulmonary disease (COPD) (*p* for interaction = 0.043), anion gap (*p* for interaction = 0.017), and serum creatinine (*p* for interaction = 0.020) significantly modified the MV–mortality association. The adverse association was substantially more pronounced in patients with an elevated anion gap (OR 6.62, 95% CI 3.28–13.38), lower serum creatinine (OR 3.73, 95% CI 1.85–7.54), and those without COPD (OR 3.23, 95% CI 2.19–4.78).

**Conclusion:**

Mechanical ventilation is independently associated with 28-day mortality in ICU sepsis patients, particularly in those with an elevated anion gap, lower serum creatinine, and without COPD. These findings highlight the importance of judicious MV use in sepsis patients and support individualized patient assessment and tailored respiratory management strategies in clinical practice.

**Clinical trial registration:**

Identifier ChiCTR2500109053 https://www.chictr.org.cn/showproj.html?proj=283223.

## 1 Introduction

Sepsis represents a major global health crisis that continues to challenge healthcare systems worldwide (1). Defined as life-threatening organ dysfunction caused by a dysregulated host response to infection, sepsis affects millions of patients annually (2). Recent epidemiological data from 2024 demonstrate that sepsis mortality remains approximately 22.5%, accounting for 19.7% of all global deaths, with an estimated 49 million cases occurring worldwide annually (3, 4). In China, a national cross-sectional survey demonstrated that 37.3% of ICU patients develop sepsis, with an associated 28-day mortality rate of 34.5% (5). More recent evidence indicates that mortality among Chinese patients with sepsis persists at 31.1% (95% CI 25.3–36.9%), underscoring the urgent need for enhanced clinical evaluation and optimized intervention strategies (6, 7).

Mechanical ventilation (MV) represents a fundamental life-support intervention that assists or replaces spontaneous breathing in critically ill patients. Its core purposes extend beyond merely improving gas exchange to include alleviating respiratory muscle fatigue, maintaining effective ventilation, and reducing respiratory workload—thereby providing crucial time for organ function recovery in sepsis patients (2, 8).

Yet, this life-sustaining intervention is inherently double-edged: while addressing hypoxemia, it simultaneously introduces adverse effects such as ventilator-associated lung injury (VALI), ventilator-associated pneumonia, and circulatory dysfunction (9). Clinical evidence indicates that approximately 48% of sepsis patients require MV during their ICU stay, with ventilated patients exhibiting a significantly elevated mortality rate (adjusted hazard ratio 2.11, 95% CI 1.58–2.82) (10, 11). These risks become particularly pronounced in specific patient populations, such as those with acute respiratory distress syndrome (ARDS), where 28-day mortality rates can reach 40–60% (11).

The relationship between MV and patient outcomes is notably complex. MV independently increases 28-day mortality in sepsis patients (adjusted OR 2.67, 95% CI 1.98–3.62), even after controlling for age, disease severity, and organ dysfunction (12). In contrast, some studies suggest MV may primarily reflect underlying illness severity rather than directly causing increased mortality when confounding is controlled rigorously (11). However, whether the mechanisms underlying this heterogeneity in the MV–mortality association stem from differences in patient profiles or contextual factors remains unclear. Building on this complexity, emerging evidence suggests that chronic obstructive pulmonary disease (COPD), anion gap (AG), and serum creatinine (SCr) may serve as critical effect modifiers in this association (13–17). COPD, a chronic inflammatory respiratory condition, fundamentally alters the clinical effect of mechanical ventilation in sepsis patients, potentially due to pre-existing respiratory compromise, altered lung mechanics, and increased susceptibility to ventilator-associated complications (18). Similarly, AG—a reliable measure of metabolic acidosis—reflects acid–base imbalances that may substantially exacerbate MV-related complications through impaired hemodynamics and tissue oxygenation (19). SCr, a key indicator of renal function, has also emerged as a vital modifying factor; elevated SCr levels reflect impaired renal clearance of metabolic byproducts, fluid accumulation, and electrolyte disturbances that frequently co-occur with sepsis and can interact profoundly with the physiological effects of positive-pressure ventilation (20, 21). Despite their potential clinical significance, how these specific clinical and laboratory parameters modify the MV–mortality association remains insufficiently investigated, particularly in Chinese ICU populations. Furthermore, the impact of MV varies considerably across patient subgroups, with early ventilation potentially benefiting patients with higher severity scores while paradoxically increasing mortality among those with milder presentations (22). The heterogeneity of study populations, clinical settings, and methodological approaches across healthcare systems further complicates efforts to define the true causal relationship between MV and mortality outcomes (23).

To address these knowledge gaps, we conducted this retrospective cohort study to comprehensively evaluate the independent association between mechanical ventilation and 28-day mortality in ICU sepsis patients. Our study spans a contemporary timeframe from January 1, 2017, to December 31, 2024, capturing the latest trends in real-world intensive care practice. A notable methodological strength is the rigorous adjustment for confounding through the inclusion of 25 clinically relevant variables—encompassing demographics, vital signs, 11 laboratory parameters, and 7 comorbidities. Using a large sample with comprehensive covariate adjustment, we aimed to clarify the precise relationship between MV and short-term mortality, and to specifically investigate how clinical characteristics such as COPD, AG, and SCr modify this association across diverse patient subgroups. The findings will provide robust, locally derived evidence to assist critical care physicians in optimizing ventilation strategies and individualizing clinical decision-making for critically ill sepsis patients.

## 2 Methods

### 2.1 Study population

**Figure 1:**
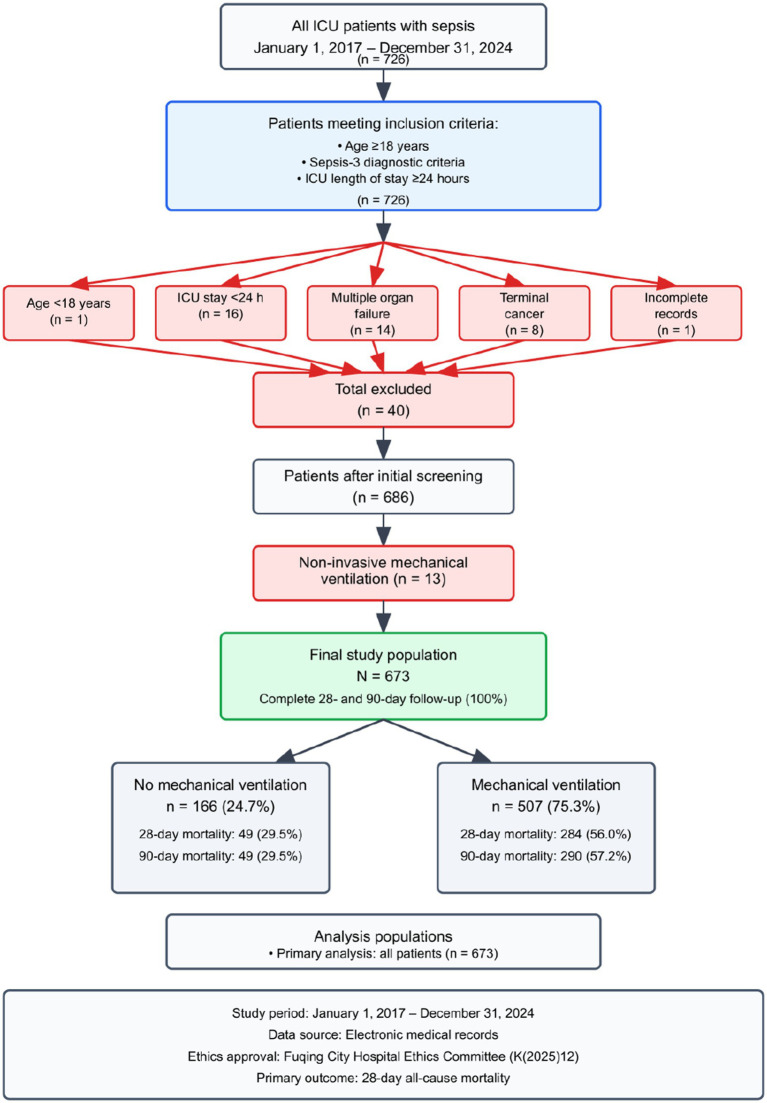
Study design and patient inclusion. Flow diagram illustrating patient selection in this retrospective cohort study of sepsis in ICU patients (January 2017–December 2024). Of 726 initially identified patients, 40 were excluded during initial screening for various reasons: age <18 years (*n* = 1), ICU stay <24 h (*n* = 16), multiple organ failure (*n* = 14), terminal cancer (*n* = 8), and incomplete records (*n* = 1). After initial screening, 686 patients remained, with an additional 13 patients excluded for receiving non-invasive mechanical ventilation. The final study population comprised 673 patients with complete follow-up: 166 patients (24.7%) without mechanical ventilation and 507 patients (75.3%) with mechanical ventilation. Mortality rates at 28 days were 29.5% (49/166) and 56.0% (284/507) in the non-mechanical ventilation and mechanical ventilation groups, respectively. The 90-day mortality rates were 29.5% (49/166) and 57.2% (290/507). Data were obtained from electronic medical records with ethics approval from Fuqing City Hospital Ethics Committee (K(2025)12).

We conducted a retrospective cohort study of adult sepsis patients admitted to the ICU of Fuqing Affiliated Hospital, Fujian Medical University (China), between January 1, 2017, and December 31, 2024, with follow-up through December 31, 2024. We defined sepsis according to Sepsis-3 criteria as life-threatening organ dysfunction caused by a dysregulated host response to infection (2). We enrolled patients aged ≥18 years who met Sepsis-3 criteria and had an ICU length of stay ≥24 h. We excluded patients with ICU stay <24 h, multiple organ failure (defined as dysfunction in ≥2 organ systems with SOFA subscore ≥2 for each involved system) (2), end-stage renal disease, advanced malignancy, or substantially missing clinical data. To maintain a homogeneous study population focused on invasive mechanical ventilation outcomes, we further excluded patients who received non-invasive ventilation (NIV) alone. After applying all inclusion and exclusion criteria, our final study cohort consisted of 673 patients (Figure 1), Assuming an adjusted OR of 2.0 for MV and 28-day mortality, a baseline mortality rate of 31.1%, *α* of 0.05, and 80% power, a minimum sample size of 600 was required, indicating our sample of 673 was sufficient.

### 2.2 Data collection and measurement methods

All data were extracted from our hospital’s electronic medical record system (HIS) by trained clinical investigators using standardized data collection forms. For demographic variables, age and sex were obtained from admission records, while BMI was calculated from height and weight measured at ICU admission.

#### 2.2.1 Clinical severity assessment

APACHE II scores were calculated using worst physiological parameters within the first 24 h of ICU admission, following standardized scoring protocols. Sepsis diagnosis was confirmed according to Sepsis-3 criteria by reviewing clinical documentation and laboratory results.

#### 2.2.2 Laboratory measurements

All laboratory parameters (lactate dehydrogenase, anion gap, serum creatinine, etc.) were obtained from the hospital’s laboratory information system, with values representing the worst measurements within 24 h of ICU admission. Specifically, the anion gap was calculated using the standard formula: Na^+^ − (Cl^−^ + HCO₃^−^), where Na^+^, Cl^−^, and HCO₃^−^ (reported as total CO₂) were measured from routine venous serum electrolyte panels on a Beckman Coulter clinical chemistry analyzer AU5800 (24). This methodology aligns with standard clinical chemistry practices for metabolic assessment (19).

#### 2.2.3 Outcome assessment

The primary outcome (28-day all-cause mortality) was ascertained through hospital records and telephone follow-up for patients discharged alive. Mortality status was independently verified by two investigators.

#### 2.2.4 Quality assurance

A random sample of 10% of cases underwent duplicate data extraction by a second investigator, with discrepancies resolved through consensus. All extracted data were cross-validated against source documents to ensure accuracy.

### 2.3 Variables

The primary exposure variable was mechanical ventilation, defined as receipt of invasive mechanical ventilation during ICU stay. MV was delivered using volume-controlled ventilation with a tidal volume of 6–8 mL/kg predicted body weight, and FiO_2_ adjusted to maintain SpO_2_ ≥ 92%, following ARDS Net guidelines (25, 26). Positive end-expiratory pressure (PEEP) was initially established at 5–8 cm H₂O and systematically titrated to achieve optimal levels through individualized assessment of respiratory mechanics parameters. Duration and timing of MV initiation varied and were not uniformly recorded. All mechanically ventilated patients in our study cohort received invasive mechanical ventilation. Non-invasive ventilation (NIV) was rarely used for sepsis in our ICU, with only 2% of screened patients receiving NIV alone. These patients were excluded to maintain a homogeneous exposure group focused on invasive MV.

The primary outcome was all-cause 28-day mortality, defined as death from any cause within 28 days of ICU admission, ascertained through hospital electronic medical records or telephone follow-up (for patients who died after discharge but within the 28-day period). This outcome was recorded as a binary variable (death = 1, survival = 0) in the database.

The secondary outcome was all-cause 90-day mortality, defined as death from any cause within 90 days of ICU admission, ascertained through the same methods as the primary outcome (hospital electronic medical records and telephone follow-up for discharged patients). This outcome was also recorded as a binary variable (death = 1, survival = 0) in the database.

Potential confounding variables included 25 prespecified covariates: demographic characteristics (age, sex, and BMI); seven comorbidities (hypertension, diabetes mellitus, chronic obstructive pulmonary disease [COPD], congestive heart failure [CHF], chronic kidney disease [CKD], chronic liver disease [CLD], and stroke); disease severity (APACHE II score); vital signs (heart rate and mean arterial pressure); 11 laboratory parameters (platelet count, international normalized ratio [INR], albumin, direct bilirubin, bicarbonate, anion gap, serum creatinine, blood urea nitrogen [BUN], lactate dehydrogenase [LDH], procalcitonin, and C-reactive protein [CRP]); and treatment variables (antibacterial therapy). Antibacterial therapy categorized as: (1) no antibiotics; (2) narrow-spectrum antibiotics (targeting a limited range of bacteria, e.g., penicillin G or first-generation cephalosporins); (3) broad-spectrum antibiotics (covering a wide range of Gram-positive and Gram-negative pathogens, including antipseudomonal agents such as piperacillin-tazobactam or carbapenems); or (4) combination antibacterial therapy (use of two or more antibiotics with different mechanisms or spectra, typically a beta-lactam plus an aminoglycoside or fluoroquinolone) (27). These covariates were selected based on established literature and pathophysiological mechanisms linking these factors to both mechanical ventilation requirements and mortality outcomes in sepsis (28, 29). Multiple variables had missing data, with BMI (32.84%), APACHE II score (11.14%), and procalcitonin (7.88%) having the highest missing rates. We handled missing data using multiple imputation with the missForest algorithm.

For subgroup and interaction analyses, we *a priori* prespecified 25 baseline characteristics, including demographics, seven comorbidities, disease severity, vital signs, 11 laboratory parameters, and antibacterial therapy (details in Section 2.3), as potential effect modifiers. The selection of these variables was fundamentally based on established pathophysiological mechanisms linking metabolic dysfunction and baseline health status to outcomes in mechanically ventilated patients. To avoid the arbitrary selection of cut-off values, 14 continuous laboratory parameters within the study cohort were categorized into tertiles based on their overall distribution. This strategy aligns with standard methodological approaches for biomarker stratification in clinical observational studies.

We conducted subgroup analyses to assess effect modification by key clinical and biochemical variables. We categorized anion gap into tertiles based on distribution [low: ≤12.8 mmol/L, middle: 12.9–16.8 mmol/L, high: ≥16.9 mmol/L] to assess metabolic acidosis status. We similarly categorized serum creatinine into tertiles [low: ≤96.0 μmol/L, middle: 96.1–174.0 μmol/L, high: ≥174.1 μmol/L], using it as both a marker of renal function and potential surrogate for muscle mass. Additionally, we examined chronic obstructive pulmonary disease (COPD) as a binary variable (present vs. absent) to evaluate its modifying effect on the association between invasive mechanical ventilation and mortality.

### 2.4 Statistical analysis

Sample size adequacy was confirmed through *post hoc* power calculations, ensuring sufficient detection of clinically meaningful effects. We presented continuous variables as mean ± standard deviation for normally distributed data or median (interquartile range) for skewed distributions, and expressed categorical variables as frequencies and percentages. We assessed normality of continuous variables using the Shapiro–Wilk test. Between-group comparisons were performed using the chi-square test for categorical variables, Student’s *t*-test for normally distributed continuous variables, or Mann–Whitney U test for non-normally distributed continuous variables.

To evaluate the independent association between mechanical ventilation and mortality outcomes, we conducted multivariable logistic regression analyses using two models: an unadjusted model including only mechanical ventilation as the exposure variable, and a fully adjusted model incorporating all 25 prespecified covariates. The adjusted model included the following variables: demographics (age, sex, BMI); comorbidities (hypertension, diabetes, COPD, CHF, CKD, CLD, stroke); disease severity indicators (APACHE II score); vital signs (heart rate, mean arterial pressure); laboratory parameters (platelet count, international normalized ratio, albumin, direct bilirubin, bicarbonate, anion gap, serum creatinine, blood urea nitrogen, lactate dehydrogenase, procalcitonin, C-reactive protein); and treatment variables (antibacterial therapy).

Subgroup analyses were performed to assess potential effect modification by stratifying according to clinically relevant variables including age, sex, BMI, comorbidities, APACHE II score, and laboratory parameters. For continuous stratification variables, we categorized them based on clinical cut-points or tertiles. Interaction terms were tested using likelihood ratio tests, with a two-sided *p*-value < 0.05 considered statistically significant. To address missing data, we employed multiple imputation using the missForest algorithm, generating five complete datasets. Results were pooled according to Rubin’s rules. All statistical analyses were performed using Python version 3.10.12, with a two-sided *p*-value < 0.05 considered statistically significant.

To address potential sources of bias in this retrospective cohort study, we implemented several measures. Selection bias was minimized through strict inclusion/exclusion criteria ensuring cohort homogeneity. Information bias from missing data was handled using multiple imputation (missForest algorithm). Confounding was addressed via multivariable logistic regression adjusting for key covariates selected based on literature. Additionally, sensitivity analyses were performed by excluding cases with >10% missing data to verify result robustness, and subgroup analyses examined potential effect modification.

## 3 Results

### 3.1 Characteristics at baseline

Table 1 presents the baseline characteristics stratified by mechanical ventilation status. Among the 673 ICU sepsis patients enrolled, 507 (75.3%) received mechanical ventilation and 166 (24.7%) did not. Mechanically ventilated patients exhibited substantially higher 28-day mortality than non-ventilated counterparts (56.0% vs. 29.5%, *p* < 0.001), representing an absolute increase in mortality rate of 26.5 percentage points and approximately 90% relative increase in mortality rate. The 90-day mortality trajectory demonstrated an identical pattern, with rates of 57.2% in the mechanical ventilation group versus 29.5% in the non-ventilation group (*p* < 0.001), confirming that this mortality disparity persisted throughout the extended follow-up period.

**Table 1:** Baseline characteristics of study participants.

Characteristics	Overall (*n* = 673)	No MV (*n* = 166)	MV (*n* = 507)	28-day Mortality *p*-value	90-day Mortality *p*-value	*p*-value*
28-day mortality						
Survived	340 (50.5%)	117 (70.5%)	223 (44.0%)	–	–	<0.001
Deceased	333 (49.5%)	49 (29.5%)	284 (56.0%)	–	–	<0.001
90-day mortality						
Survived	334 (49.6%)	117 (70.5%)	217 (42.8%)	–	–	<0.001
Deceased	339 (50.4%)	49 (29.5%)	290 (57.2%)	–	–	<0.001
Demographics						
Age, years	70.25 ± 14.17	67.96 ± 14.57	71.00 ± 13.97	<0.001	<0.001	0.016
Sex						
Male	422 (62.7%)	98 (59.0%)	324 (63.9%)	0.898	0.698	0.301
Female	251 (37.3%)	68 (41.0%)	183 (36.1%)	0.898	0.698	0.301
BMI, kg/m^2^	22.27 ± 4.21	21.95 ± 4.33	22.38 ± 4.17	0.766	0.693	0.250
Comorbidities						
Diabetes mellitus						
No	438 (65.1%)	97 (58.4%)	341 (67.3%)	0.964	0.558	0.048
Yes	235 (34.9%)	69 (41.6%)	166 (32.7%)	0.964	0.558	0.048
Hypertension						
No	390 (57.9%)	96 (57.8%)	294 (58.0%)	0.643	0.487	1.000
Yes	283 (42.1%)	70 (42.2%)	213 (42.0%)	0.643	0.487	1.000
Chronic obstructive pulmonary disease						
No	630 (93.6%)	161 (97.0%)	469 (92.5%)	0.074	0.096	0.062
Yes	43 (6.4%)	5 (3.0%)	38 (7.5%)	0.074	0.096	0.062
Congestive heart failure						
No	556 (82.6%)	132 (79.5%)	424 (83.6%)	0.100	0.008	0.273
Yes	117 (17.4%)	34 (20.5%)	83 (16.4%)	0.100	0.008	0.273
Chronic kidney disease						
No	481 (71.5%)	119 (71.7%)	362 (71.4%)	0.011	0.002	1.000
Yes	192 (28.5%)	47 (28.3%)	145 (28.6%)	0.011	0.002	1.000
Chronic liver disease						
No	666 (99.0%)	164 (98.8%)	502 (99.0%)	0.260	0.279	1.000
Yes	7 (1.0%)	2 (1.2%)	5 (1.0%)	0.260	0.279	1.000
Stroke						
No	572 (85.0%)	146 (88.0%)	426 (84.0%)	0.194	0.187	0.269
Yes	101 (15.0%)	20 (12.0%)	81 (16.0%)	0.194	0.187	0.269
Clinical severity and vital signs						
APACHE II	22.72 ± 7.23	18.30 ± 6.95	24.16 ± 6.72	<0.001	<0.001	<0.001
Heart rate, bpm	108.23 ± 23.19	103.25 ± 21.87	109.86 ± 23.40	<0.001	<0.001	0.001
Mean arterial pressure, mmHg	85.54 ± 18.86	83.68 ± 19.15	86.15 ± 18.74	0.128	0.350	0.142
Laboratory parameters						
Platelet count, ×10^9^/L	145.00 (84.00–218.00)	125.50 (76.25–208.50)	148.00 (87.50–221.00)	<0.001	<0.001	0.654
International normalized ratio	1.46 ± 0.48	1.38 ± 0.39	1.49 ± 0.50	<0.001	<0.001	0.011
Albumin, g/L	25.62 ± 6.40	27.07 ± 5.47	25.15 ± 6.61	0.032	0.137	<0.001
Direct bilirubin, μmol/L	7.90 (4.60–16.90)	7.70 (4.33–16.93)	8.10 (4.60–16.90)	0.002	0.012	0.766
Bicarbonate, mmol/L	19.18 ± 5.21	18.61 ± 5.39	19.37 ± 5.15	0.175	0.389	0.100
Anion gap, mmol/L	15.24 ± 5.75	15.00 ± 5.11	15.32 ± 5.94	<0.001	<0.001	0.536
Serum creatinine, μmol/L	132.00 (86.00–217.00)	131.50 (79.75–208.50)	133.00 (86.50–223.50)	0.028	0.005	0.982
Blood urea nitrogen, mmol/L	11.90 (7.70–18.60)	12.15 (7.17–19.50)	11.70 (7.95–18.30)	<0.001	<0.001	0.717
Lactate dehydrogenase, IU/L	277.00 (199.00–478.00)	260.50 (204.00–365.75)	289.00 (199.00–527.50)	<0.001	<0.001	0.003
Procalcitonin, ng/mL	38.69 (5.24–100.00)	41.01 (2.85–100.00)	37.97 (5.86–100.00)	0.619	0.411	0.948
C-reactive protein, mg/L	155.75 (71.86–200.00)	181.15 (84.45–200.00)	147.66 (67.84–200.00)	0.641	0.383	0.018
Treatment						
Antibacterial therapy						
No antibacterial	19 (2.8%)	6 (3.6%)	13 (2.6%)	0.028	0.072	0.028
Narrow-spectrum antibiotics	39 (5.8%)	8 (4.8%)	31 (6.1%)	0.034	0.072	0.028
Broad-spectrum antibiotics	469 (69.7%)	129 (77.7%)	340 (67.1%)	0.011	0.011	0.028
Combination antibacterial therapy	146 (21.7%)	23 (13.9%)	123 (24.3%)	0.054	0.083	0.028

* Indicates statistical significance at *p* < 0.05.

Data are expressed as mean ± standard deviation (SD) for normally distributed continuous variables, median [interquartile range (IQR)] for non-normally distributed variables, or frequency (percentage) for categorical variables. Comparisons between groups were performed using Student’s *t*-test, Mann–Whitney U test, Pearson’s *χ*^2^ test, or Fisher’s exact test, as appropriate. Missing data for body mass index (32.8%), APACHE II (11.1%), and procalcitonin (7.9%) were handled using multiple imputation based on the missForest algorithm; all other variables had negligible missing data (<2%). No patients were lost to follow-up during the 28-day or 90-day observation periods.

BMI, body mass index; COPD, chronic obstructive pulmonary disease; CHF, congestive heart failure; CKD, chronic kidney disease; CLD, chronic liver disease; APACHE II, Acute Physiology and Chronic Health Evaluation II; MAP, mean arterial pressure; PLT, platelets; INR, international normalized ratio; DBIL, direct bilirubin; AG, anion gap; SCr, serum creatinine; BUN, blood urea nitrogen; LDH, lactate dehydrogenase; PCT, procalcitonin; CRP, C-reactive protein.

Several clinically relevant differences emerged between groups at baseline. Mechanically ventilated patients presented with higher disease severity, as reflected in APACHE II scores (24.16 ± 6.72 vs. 18.30 ± 6.95, *p* < 0.001), advanced age (71.00 ± 13.97 vs. 67.96 ± 14.57 years, *p* = 0.016), and lower serum albumin levels (25.15 ± 6.61 vs. 27.07 ± 5.47 g/L, *p* < 0.001). These findings point to disease severity and nutritional status as potential confounding variables requiring adjustment in downstream analyses.

Regarding comorbidity burden, the analysis revealed that major chronic conditions were generally well-balanced between groups. Chronic obstructive pulmonary disease showed a numerically higher prevalence in the mechanical ventilation group (7.5% vs. 3.0%, *p* = 0.062), though this difference did not reach statistical significance. Hypertension (42.0% vs. 42.2%, *p* = 1.000), congestive heart failure (16.4% vs. 20.5%, *p* = 0.273), chronic kidney disease (28.6% vs. 28.3%, *p* = 1.000), chronic liver disease (1.0% vs. 1.2%, *p* = 1.000), and stroke history (16.0% vs. 12.0%, *p* = 0.269) demonstrated comparable prevalence rates across both groups. These balanced distributions suggest that major comorbidities were unlikely to confound the association between mechanical ventilation and mortality. Paradoxically, diabetes mellitus showed higher prevalence in the non-ventilation group (41.6% vs. 32.7%, *p* = 0.048), yet this population maintained substantially lower mortality than mechanically ventilated patients—a finding that further underscores the strength of the mechanical ventilation–mortality association. Antibacterial treatment strategies differed significantly between groups (*p* = 0.028), with combination therapy more prevalent in the mechanical ventilation cohort (24.3% vs. 13.9%), likely reflecting disease complexity and infection severity in ventilated patients. Notably, 19 patients (2.8%) received no antibacterial agents; these cases were managed based on suspected viral respiratory infections (e.g., Influenza, COVID-19) or invasive fungal infections without evidence of bacterial coinfection, consistent with current antimicrobial stewardship guidelines advocating restriction of antibacterial use in confirmed non-bacterial sepsis (26, 30).

### 3.2 Univariate analysis

Univariate logistic regression revealed mechanical ventilation as the strongest factor associated with mortality in the sepsis cohort (Table 2). Mechanically ventilated patients demonstrated substantially higher odds of 28-day mortality (OR 3.04, 95% CI 2.09–4.43, *p* < 0.001) and 90-day mortality (OR 3.19, 95% CI 2.19–4.65, *p* < 0.001) compared to non-ventilated patients.

**Table 2:** Patient characteristics and univariate analysis for 28-day and 90-day mortality.

Variables	Overall (*n* = 673)	OR (95% CI) 28-day	*p*-value 28-day	OR (95% CI) 90-day	*p*-value 90-day
Age, years	70.25 ± 14.17	1.02 (1.01, 1.03)	<0.001	1.02 (1.01, 1.03)	<0.001
Sex					
Male	422 (62.7%)	1.00 (Reference)	–	1.00 (Reference)	–
Female	251 (37.3%)	1.02 (0.75, 1.40)	0.898	0.94 (0.69, 1.28)	0.698
BMI, kg/m^2^	22.27 ± 4.21	1.01 (0.96, 1.05)	0.766	1.01 (0.97, 1.05)	0.693
Diabetes mellitus					
No	438 (65.1%)	1.00 (Reference)	–	1.00 (Reference)	–
Yes	235 (34.9%)	0.99 (0.72, 1.36)	0.964	1.10 (0.80, 1.51)	0.558
Hypertension					
No	390 (57.9%)	1.00 (Reference)	–	1.00 (Reference)	–
Yes	283 (42.1%)	1.07 (0.79, 1.46)	0.643	1.11 (0.82, 1.51)	0.487
Chronic obstructive pulmonary disease					
No	630 (93.6%)	1.00 (Reference)	–	1.00 (Reference)	–
Yes	43 (6.4%)	1.79 (0.94, 3.38)	0.074	1.72 (0.91, 3.25)	0.096
Congestive heart failure					
No	556 (82.6%)	1.00 (Reference)	–	1.00 (Reference)	–
Yes	117 (17.4%)	1.40 (0.94, 2.09)	0.100	1.73 (1.15, 2.60)	0.008
Chronic kidney disease					
No	481 (71.5%)	1.00 (Reference)	–	1.00 (Reference)	–
Yes	192 (28.5%)	1.55 (1.11, 2.17)	0.011	1.71 (1.22, 2.41)	0.002
Chronic liver disease					
No	666 (99.0%)	1.00 (Reference)	–	1.00 (Reference)	–
Yes	7 (1.0%)	2.58 (0.50, 13.37)	0.260	2.48 (0.48, 12.90)	0.279
Stroke					
No	572 (85.0%)	1.00 (Reference)	–	1.00 (Reference)	–
Yes	101 (15.0%)	1.32 (0.87, 2.03)	0.194	1.33 (0.87, 2.04)	0.187
APACHE II	22.72 ± 7.23	1.05 (1.03, 1.08)	<0.001	1.05 (1.03, 1.08)	<0.001
Heart rate, bpm	108.23 ± 23.19	1.01 (1.01, 1.02)	<0.001	1.01 (1.01, 1.02)	<0.001
Mean arterial pressure, mmHg	85.54 ± 18.86	0.99 (0.99, 1.00)	0.128	1.00 (0.99, 1.00)	0.350
Platelet count, ×10^9^/L	145.00 (84.00–218.00)	1.00 (1.00, 1.00)	<0.001	1.00 (1.00, 1.00)	<0.001
International normalized ratio	1.46 ± 0.48	2.20 (1.54, 3.14)	<0.001	2.02 (1.42, 2.86)	<0.001
Albumin, g/L	25.62 ± 6.40	0.97 (0.95, 1.00)	0.032	0.98 (0.96, 1.01)	0.137
Direct bilirubin, μmol/L	7.90 (4.60–16.90)	1.01 (1.00, 1.01)	0.002	1.01 (1.00, 1.01)	0.012
Bicarbonate, mmol/L	19.18 ± 5.21	0.98 (0.95, 1.01)	0.175	0.99 (0.96, 1.02)	0.389
Anion gap, mmol/L	15.24 ± 5.75	1.07 (1.04, 1.11)	<0.001	1.07 (1.04, 1.11)	<0.001
Serum creatinine, μmol/L	132.00 (86.00–217.00)	1.00 (1.00, 1.00)	0.028	1.00 (1.00, 1.00)	0.005
Blood urea nitrogen, mmol/L	11.90 (7.70–18.60)	1.03 (1.02, 1.05)	<0.001	1.03 (1.02, 1.05)	<0.001
Lactate dehydrogenase, IU/L	277.00 (199.00–478.00)	1.00 (1.00, 1.00)	<0.001	1.00 (1.00, 1.00)	<0.001
Procalcitonin, ng/mL	38.69 (5.24–100.00)	1.00 (1.00, 1.00)	0.619	1.00 (1.00, 1.00)	0.411
C-reactive protein, mg/L	155.75 (71.86–200.00)	1.00 (1.00, 1.00)	0.641	1.00 (1.00, 1.00)	0.383
Mechanical ventilation					
No	166 (24.7%)	1.00 (Reference)	–	1.00 (Reference)	–
Yes	507 (75.3%)	3.04 (2.09, 4.43)	<0.001	3.19 (2.19, 4.65)	<0.001
Antibacterial therapy					
No antibacterial	19 (2.8%)	1.00 (Reference)	–	1.00 (Reference)	–
Narrow-spectrum antibiotics	39 (5.8%)	0.25 (0.07, 0.90)	0.034	0.31 (0.09, 1.11)	0.072
Broad-spectrum antibiotics	469 (69.7%)	0.23 (0.08, 0.71)	0.011	0.23 (0.08, 0.71)	0.011
Combination antibacterial therapy	146 (21.7%)	0.32 (0.10, 1.02)	0.054	0.36 (0.11, 1.14)	0.083

Data are presented as mean ± standard deviation (SD) for normally distributed continuous variables, median [interquartile range, IQR] for non-normally distributed variables, and number (percentage) for categorical variables. Univariate logistic regression models were used to estimate odds ratios (OR) and 95% confidence intervals (CI) for 28-day and 90-day mortality. For categorical variables, the reference group was defined as the absence of the condition (e.g., “No”) or the first category listed. A two-sided *p* value < 0.05 was considered statistically significant. In accordance with the journal’s two-decimal formatting guidelines, ORs and 95% CIs are rounded to 1.00. For precise, unrounded values, please refer to Supplementary Table S9.

OR, odds ratio; CI, confidence interval; MV, mechanical ventilation; BMI, body mass index; DM, diabetes mellitus; HTN, hypertension; COPD, chronic obstructive pulmonary disease; CHF, congestive heart failure; CKD, chronic kidney disease; CLD, chronic liver disease; APACHE II, Acute Physiology and Chronic Health Evaluation II; MAP, mean arterial pressure; PLT, platelets; INR, international normalized ratio; DBIL, direct bilirubin; AG, anion gap; SCr, serum creatinine; BUN, blood urea nitrogen; LDH, lactate dehydrogenase; PCT, procalcitonin; CRP, C-reactive protein.

International normalized ratio (INR) emerged as the second strongest factor associated with mortality. Each unit increase in INR corresponded to 120% higher odds of 28-day mortality (OR 2.20, 95% CI 1.54–3.14, *p* < 0.001) and 102% higher odds of 90-day mortality (OR 2.02, 95% CI 1.42–2.86, *p* < 0.001), underscoring the importance of coagulation dysfunction in sepsis outcomes. Advancing age and disease severity, as measured by APACHE II score, independently associated with mortality at both time points (28-day age: OR 1.02, 95% CI 1.01–1.03; 90-day age: OR 1.02, 95% CI 1.01–1.03; both *p* < 0.001; APACHE II: OR 1.05, 95% CI 1.03–1.08 for both endpoints, both *p* < 0.001). Each 1 mmol/L increase in anion gap—a marker of metabolic acidosis—associated with 7% higher odds of mortality (OR 1.07, 95% CI 1.04–1.11, *p* < 0.001). Among renal function parameters, each 1 mmol/L increase in blood urea nitrogen associated with 3% higher odds of mortality (OR 1.03, 95% CI 1.02–1.05, *p* < 0.001).

The extended evaluation of comorbid conditions revealed important associations. Chronic kidney disease significantly associated with increased mortality at both 28 days (OR 1.55, 95% CI 1.11–2.17, *p* = 0.011) and 90 days (OR 1.71, 95% CI 1.22–2.41, *p* = 0.002). Notably, congestive heart failure did not significantly associate with 28-day mortality (*p* = 0.100) but demonstrated a robust association with 90-day mortality (OR 1.73, 95% CI 1.15–2.60, *p* = 0.008), suggesting progressive hemodynamic impacts over extended follow-up. Other major comorbidities—chronic obstructive pulmonary disease, chronic liver disease, and stroke history—showed no statistically significant associations with mortality.

Antibacterial therapy demonstrated strong protective effects. Compared with no antibacterial therapy, broad-spectrum antibiotics associated with 77% lower 28-day mortality odds (OR 0.23, 95% CI 0.08–0.71, *p* = 0.011), and narrow-spectrum antibiotics with 75% lower odds (OR 0.25, 95% CI 0.07–0.90, *p* = 0.034), highlighting the critical importance of appropriate early antimicrobial initiation. Each 1 g/L increase in serum albumin associated with 3% lower odds of 28-day mortality (OR 0.97, 95% CI 0.95–1.00, *p* = 0.032), reflecting the protective effect of adequate nutritional status. Demographic and conventional cardiovascular factors—including sex, body mass index, diabetes mellitus, and hypertension—demonstrated no significant associations with mortality (all *p* > 0.05).

### 3.3 Multivariable regression analysis

Multivariable logistic regression confirmed that mechanical ventilation remained independently associated with elevated mortality in sepsis patients following comprehensive adjustment (Table 3). In the unadjusted analysis, mechanical ventilation strongly associated with 28-day mortality (OR 3.04, 95% CI 2.09–4.43, *p* < 0.001). After comprehensive adjustment for all 25 prespecified covariates—encompassing demographics, disease severity, 7 comorbidities, vital signs, 11 laboratory parameters, and antibacterial therapy—this association remained highly significant (adjusted OR 2.62, 95% CI 1.67–4.11, *p* < 0.001), with only moderate attenuation in the effect estimate. This adjusted association corresponded to 162% higher odds of 28-day mortality in mechanically ventilated versus non-ventilated patients. Similarly, mechanical ventilation maintained statistical significance for 90-day mortality in both the unadjusted model (OR 3.19, 95% CI 2.19–4.65, *p* < 0.001) and the fully adjusted model (adjusted OR 2.77, 95% CI 1.77–4.34, *p* < 0.001), with approximately 13% attenuation in the odds ratio. The adjusted 90-day estimate corresponded to 177% higher mortality odds in the ventilated population.

**Table 3:** Multivariable regression analysis of association between mechanical ventilation and 28-day and 90-day mortality.

Outcome	Model	OR (95% CI)	*p*-value
28-day mortality	Unadjusted	3.04 (2.09, 4.43)	<0.001
	Adjusted*	2.62 (1.67, 4.11)	<0.001
90-day mortality	Unadjusted	3.19 (2.19, 4.65)	<0.001
	Adjusted*	2.77 (1.77, 4.34)	<0.001

* Indicates adjustment for all 25 prespecified covariates.

Data Analysis: Multivariable logistic regression models were used to estimate adjusted odds ratios (aOR) and 95% confidence intervals (CI). The reference group for mechanical ventilation was “No mechanical ventilation.” Missing data were handled using multiple imputation by chained equations (MICE) with 20 imputed datasets. Results were pooled using Rubin’s rules.

Adjustments: The models were adjusted for the following covariates: Demographics: Age, sex, and body mass index (BMI). Comorbidities: Diabetes mellitus (DM), hypertension (HTN), chronic obstructive pulmonary disease (COPD), congestive heart failure (CHF), chronic kidney disease (CKD), chronic liver disease (CLD), and Stroke. Clinical Severity: APACHE II. Vital Signs & Laboratory Parameters: Heart rate, mean arterial pressure, platelet count, international normalized ratio (INR), albumin, direct bilirubin, bicarbonate, anion gap, serum creatinine, blood urea nitrogen, lactate dehydrogenase (LDH), procalcitonin, and C-reactive protein (CRP). Treatment: Antibacterial therapy.

Statistical Diagnostics: Missing data were handled using multiple imputation with the missForest algorithm; 5 imputed datasets were generated and pooled using Rubin’s rules. The variance inflation factor (VIF) for all covariates was <2.0, indicating the absence of multicollinearity.

Notably, the independent association of mechanical ventilation with mortality demonstrated sustained and slightly more pronounced effects during extended follow-up, emphasizing the clinical importance of close monitoring and long-term evaluation of septic patients requiring mechanical ventilation. Fundamentally, these findings establish that mechanical ventilation associates independently with both short- and long-term mortality in sepsis, an association that persists well beyond what would be expected from baseline disease severity and comorbid status alone.

### 3.4 Ventilation–mortality effect modifiers

**Figure 2:**
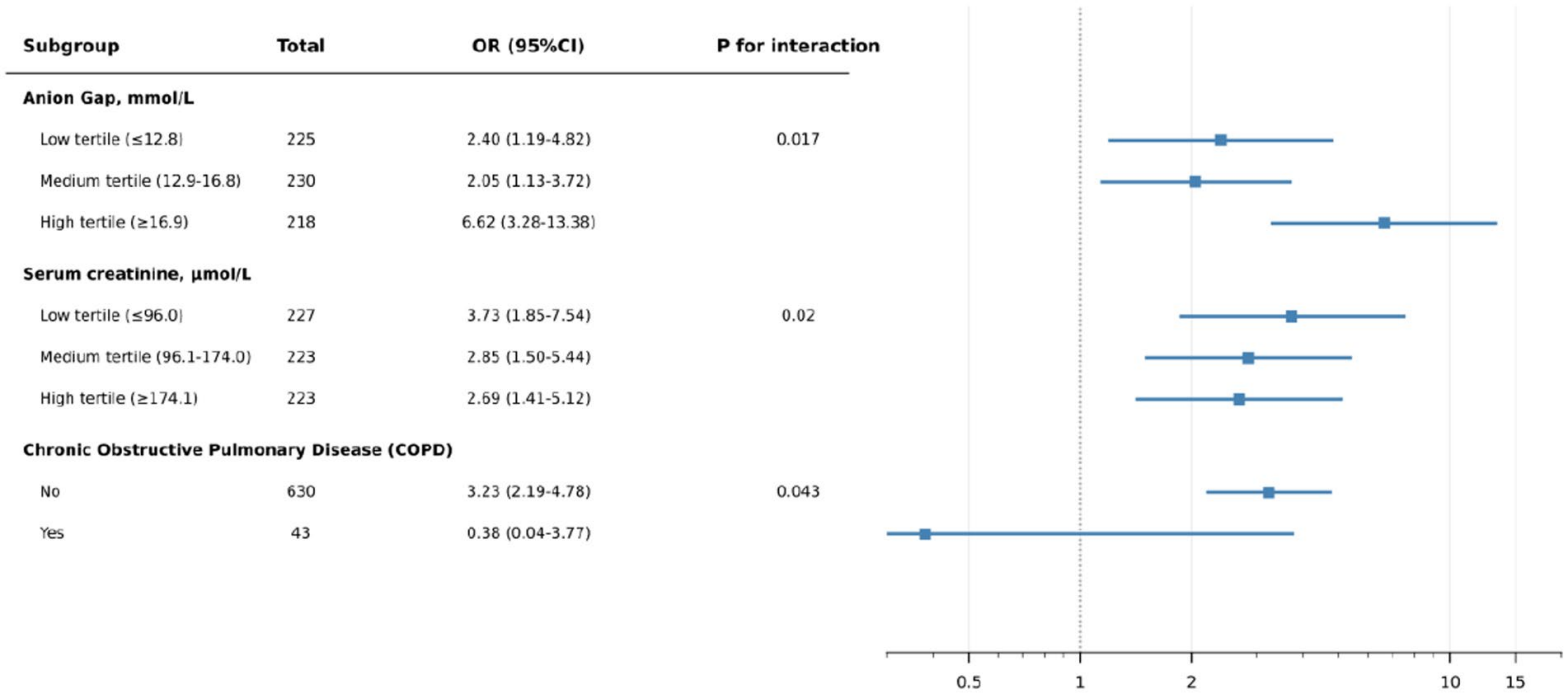
Effect modification by COPD, anion gap, and serum creatinine on the association between invasive mechanical ventilation and 28-day mortality. Adjusted for age, sex, BMI, APACHE II score, comorbidities (hypertension, diabetes, COPD, CHF, CKD, CLD, stroke), heart rate, MAP, platelet count, INR, albumin, direct bilirubin, HCO_3_^−^, anion gap, creatinine, BUN, LDH, procalcitonin, CRP, and antibacterial therapy. AG and SCr were categorized into tertiles based on the distribution of the study cohort (cutpoints shown in Methods).

Based on the fully adjusted model incorporating all 25 prespecified covariates, Figure 2 illustrates effect modification of the mechanical ventilation–mortality association across patient subgroups. Subgroup analyses revealed substantial heterogeneity in mechanical ventilation’s clinical impact across different patient characteristics, emphasizing the need for individualized therapeutic assessment.

Anion gap significantly modified this association (*p*_interaction_ = 0.017). Patients with elevated anion gap experienced markedly stronger adverse effects from mechanical ventilation (OR = 6.62, 95% CI: 3.28–13.38), compared with those in the low (OR = 2.40, 95% CI: 1.19–4.82) or medium tertile (OR = 2.05, 95% CI: 1.13–3.72). This pattern suggests that Non-invasive ventilation (NIV) was rarely metabolic acidosis substantially amplifies mechanical ventilation’s adverse prognostic impact.

Serum creatinine levels also demonstrated significant effect modification (*p*_interaction_ = 0.020). The association between mechanical ventilation and mortality was most pronounced among patients with lower serum creatinine levels (OR = 3.73, 95% CI: 1.85–7.54), gradually attenuated in the medium tertile (OR = 2.85, 95% CI: 1.50–5.44), and further diminished in those with higher levels (OR = 2.69, 95% CI: 1.41–5.12). This inverse pattern reflects complex interplay between baseline renal function and mechanical ventilation outcomes.

Notably, chronic obstructive pulmonary disease (COPD) emerged as a crucial effect modifier (*p_interaction_* = 0.043). In patients without COPD, mechanical ventilation robustly associated with increased mortality (OR = 3.23, 95% CI: 2.19–4.78); however, among patients with concomitant COPD, we observed no significant adverse association, with the confidence interval encompassing unity (OR = 0.38, 95% CI: 0.04–3.77). This distinction points to patients with preexisting chronic pulmonary compromise potentially tolerating mechanical ventilation differently or exhibiting distinct pathophysiological responses compared with those without such conditions.

Stratified 90-day analyses demonstrated consistency with 28-day findings. Anion gap maintained its effect modification role (*p*_interaction_ = 0.012), while COPD status also demonstrated persistent effect modification (*p*_interaction_ = 0.041), confirming that these interactions persist throughout extended follow-up. In contrast, the serum creatinine interaction attenuated over the extended observation period.

Collectively, these findings underscore substantial heterogeneity in the mechanical ventilation–mortality association across patient subgroups defined by metabolic status, renal function, and pulmonary comorbidity. These results highlight the clinical utility of considering anion gap, serum creatinine levels, and COPD status when evaluating mechanical ventilation’s benefit-harm profile in sepsis patients, potentially supporting more refined clinical decision-making and patient stratification strategies.

## 4 Discussion

### 4.1 Principal findings

In this retrospective cohort study involving 673 ICU patients with sepsis, we demonstrated that mechanical ventilation was independently associated with higher mortality, with an adjusted odds ratio of 2.62 (95% CI 1.67–4.11, *p* < 0.001) for 28-day mortality. Importantly, this association remained highly robust even after comprehensive adjustment for 25 clinically relevant variables (encompassing demographics, disease severity, 7 comorbidities, vital signs, 11 laboratory parameters, and antibacterial therapy). This adverse relationship persisted at the 90-day follow-up (adjusted OR 2.77, 95% CI 1.77–4.34, *p* < 0.001), confirming the durability of this relationship.

Our stratified analyses revealed significant effect modification by specific laboratory parameters and COPD. The adverse association was most significantly amplified in patients with an elevated anion gap (highest tertile OR = 6.62, 95% CI 3.28–13.38; *p_interaction_* = 0.017) and lower serum creatinine levels (lowest tertile OR = 3.73, 95% CI 1.85–7.54; *p_interaction_* = 0.020). Conversely, the presence of COPD significantly modified this relationship, with mechanical ventilation exhibiting starkly contrasting associations with mortality in patients with versus without COPD (OR = 0.38, 95% CI 0.04–3.77 versus OR = 3.23, 95% CI 2.19–4.78; *p_interaction_ =* 0.043). These interaction patterns were largely consistent in the 90-day analysis for anion gap and COPD, confirming that these effect modifications persist over extended follow-up.

### 4.2 Possible explanations and mechanisms

The association between mechanical ventilation and higher mortality in sepsis likely reflects a “double-hit” pathophysiological model (31, 32). In this model, ventilation-induced stress compounds preexisting sepsis-related systemic inflammation, primarily through ventilator-associated lung injury (VALI) triggering inflammatory cascades via mechanotransduction pathways (33). Our study advances this understanding by elucidating how distinct clinical phenotypes modify this baseline vulnerability.

With respect to our interaction analysis, we observed that severe metabolic acidosis (highest anion gap tertile) most prominently amplified this adverse association. An elevated anion gap reflects the accumulation of unmeasured anions, a state known to impair myocardial contractility and compromise vascular responsiveness (34). When subjected to positive pressure ventilation, these acidotic patients exhibit heightened vulnerability to severe hemodynamic perturbations, thereby accounting for the magnified adverse association (35, 36).

Critically, the pronounced effect modification observed in patients with lower serum creatinine (lowest tertile) operates through two distinct but complementary clinical pathways. First, low creatinine in hyperdynamic septic patients often reflects augmented renal clearance (ARC) (37). Without proactive dose individualization, ARC facilitates the rapid elimination of renally cleared antibacterials, resulting in subtherapeutic exposure and therapeutic failure (38). Second, low serum creatinine serves as a well-established clinical surrogate for reduced skeletal muscle mass (sarcopenia) (39). This preexisting physical frailty directly predisposes patients to ventilator-induced diaphragmatic dysfunction and difficult liberation, thereby linking low muscle reserve to prolonged ventilation and higher mortality (40).

Conversely, the presence of concomitant COPD significantly modified this relationship, demonstrating a stark contrast: mechanical ventilation exhibited a substantially attenuated association with mortality in this subgroup (OR = 0.38). Rather than implying a protective biological mechanism, we propose that this clinical paradox reflects divergent indications for intubation. Sepsis patients without COPD typically require mechanical ventilation for devastating systemic insults, such as severe acute respiratory distress syndrome (ARDS) or refractory shock. In contrast, COPD patients frequently present with acute hypercapnic respiratory failure secondary to respiratory muscle fatigue or bronchospasm—conditions that are generally more amenable to prompt ventilatory support. Furthermore, recognizing the inherent pulmonary vulnerability of COPD patients, clinicians often preferentially apply highly tailored, lung-protective ventilatory strategies, thereby potentially mitigating the superimposition of severe VALI.

### 4.3 Comparison with existing literature

Our adjusted odds ratio of 2.62 (95% CI 1.67–4.11) demonstrates a substantial adverse association between mechanical ventilation and mortality in sepsis patients, which aligns with existing literature documenting higher mortality in mechanically ventilated septic populations. Liu et al. reported that mechanically ventilated sepsis patients exhibited significantly higher hospital mortality compared to non-ventilated patients (OR 1.6, 95% CI 1.49–1.75) in their comprehensive analysis of 5,783 patients from the MIMIC-III database (10). Kim et al. (28) examined ventilation timing effects in a multicenter Korean cohort of 2,527 sepsis patients, observing that early mechanical ventilation was associated with lower ICU mortality compared to delayed ventilation (36.3% vs. 46.4%; OR 0.66, 95% CI 0.47–0.93). Our higher effect estimate likely reflects several methodological advantages: (1) specific focus on ventilation as the primary exposure variable; (2) extended 90-day follow-up providing comprehensive mortality assessment; (3) the rigorous robustness of our model, featuring comprehensive adjustment for 25 clinically relevant variables (including demographics, vital signs, 11 laboratory parameters, and 7 comorbidities); and (4) the examination of novel effect modifiers—specifically anion gap, serum creatinine, and COPD—of the ventilation-mortality relationship.

An elevated anion gap (≥16.9 mmol/L) demonstrated a significant interaction with mechanical ventilation on mortality (*p_interaction_* = 0.017), which is consistent with recent findings that a similar threshold (≥17.0 mmol/L) is independently associated with higher short-term mortality in septic patients with pulmonary hypertension (OR: 2.35, 95% CI: 1.68–3.29) (41, 42). This consistency across distinct critical care populations supports an anion gap around 17.0 mmol/L as a clinically relevant threshold for metabolic derangement that amplifies the adverse association of mechanical ventilation (43). Our updated analysis further revealed that variations in serum creatinine (*p_interaction_* = 0.020) and the presence of COPD (*p_interaction_* = 0.043) also exhibited profound effect modification. While previous broad cohorts like Liu et al. established the baseline vulnerability of ventilated sepsis patients, we add critical granularity (44). Our findings demonstrate that distinct clinical phenotypes—encompassing metabolic, renal, and chronic respiratory status—act synergistically to modify clinical trajectories. These nuanced interactions, which previous studies had not documented in generalized mechanically ventilated septic cohorts, underscore the necessity of moving beyond “one-size-fits-all” patient stratification.

### 4.4 Clinical implications

Our findings, robustly adjusted for 25 clinically relevant variables (including demographics, vital signs, 11 laboratory parameters, and 7 comorbidities), provide actionable insights for sepsis management. Clinicians can adopt more precise and individualized treatment strategies, potentially benefiting from incorporating anion gap, serum creatinine, and COPD status into clinical evaluations. Specifically, patients with an elevated anion gap exhibit magnified vulnerability to ventilation-associated adverse outcomes. For these individuals, careful evaluation of alternative respiratory support modalities is warranted when feasible, aligning with Surviving Sepsis Campaign 2021 guidelines emphasizing individualized respiratory support strategies (Recommendation 1B) (45). When mechanical ventilation becomes indispensable, we emphasize lung-protective strategies, such as lower tidal volumes and limited driving pressure, as crucial. Concurrently, clinicians should prioritize the correction of metabolic acidosis to help preserve hemodynamic stability and cellular energetics in this metabolically vulnerable subgroup.

Furthermore, the pronounced effect modification by low serum creatinine reveals two potential clinical targets. First, since low creatinine may reflect augmented renal clearance, clinicians should maintain heightened vigilance for subtherapeutic antibacterial exposure and consider therapeutic drug monitoring (46). Second, recognizing the clinical association between low creatinine and sarcopenia, implementing early mobilization and diaphragm-protective strategies could prove vital to preserve both skeletal and respiratory muscle reserves (17, 47). Conversely, for septic patients with concomitant COPD, clinicians should acknowledge their specific respiratory mechanics and apply highly tailored ventilatory settings that avoid dynamic hyperinflation (18, 48). Ultimately, we emphasize the value of future clinical trials evaluating tailored ventilation and pharmacological protocols for sepsis patients with distinct metabolic, renal, and chronic respiratory phenotypes.

### 4.5 Limitations

The primary clinical value of this research derives from its robust evaluation of mechanical ventilation in sepsis, uniquely strengthened by rigorous adjustment for 25 clinically relevant variables (including demographics, vital signs, 11 laboratory parameters, and 7 comorbidities). By delineating specific clinical phenotypes—particularly the pronounced effect modifications by concurrent COPD, elevated anion gap, and altered serum creatinine—we provide actionable evidence to support tailored respiratory and metabolic interventions in clinical practice. These insights illuminate promising directions for future prospective trials evaluating individualized care. Despite the methodological strengths of this study, several limitations warrant careful consideration.

First, based on our prespecified inclusion and exclusion criteria, this study excluded patients with an ICU length of stay under 24 h, as well as those presenting with preexisting multiple organ failure, end-stage renal disease, and advanced malignancies. Consequently, our findings cannot be extrapolated to these specific critically ill subpopulations. Second, this research was conducted at a single center; therefore, the broad generalizability of the findings is limited, necessitating validation through additional comparable multicenter studies. Third, the primary study population consisted of Chinese individuals; therefore, caution is required when applying our conclusions to other distinct ethnic groups, given potential variations in genetics and healthcare delivery. Fourth, inherent to all observational designs, this study can only establish an association between mechanical ventilation and mortality, rather than establish causality.

Notably, while our analysis identified COPD, anion gap, and serum creatinine as significant effect modifiers, the relatively small number of patients with COPD (*n* = 43, 6.4%) may limit the precision of effect estimates in this subgroup, warranting cautious interpretation of the observed paradoxical protective association. Finally, our multivariable models could only adjust for measurable confounding variables, leaving the potential for residual confounding from unmeasured factors. Specifically, our binary classification of mechanical ventilation did not differentiate between exact ventilation durations or specific ventilatory modes. This represents a critical unmeasured confounder, since the duration of invasive support is closely linked to subsequent complications such as ventilator-associated pneumonia and diaphragmatic dysfunction. Furthermore, different ventilation strategies—such as ultra-protective lung ventilation versus conventional modes—exert profoundly different effects on pulmonary mechanics and systemic inflammation, thereby leading to substantial variations in clinical outcomes. Additionally, the multiple imputation approach for missing data, while methodologically sound, assumes that data are missing at random, an assumption that cannot be fully verified in observational studies.

## 5 Conclusion

This single-center retrospective cohort demonstrates that invasive mechanical ventilation is independently associated with increased 28-day mortality in ICU sepsis patients. Notably, concomitant COPD status, lower serum creatinine, and elevated anion gap emerge as significant effect modifiers that amplify this adverse association, particularly in patients without COPD, with reduced renal function, or with severe metabolic acidosis. These findings illuminate the complex interactions between mechanical ventilation and distinct clinical phenotypes; we propose that preexisting respiratory compromise, altered renal-muscular baseline, and metabolic acidosis collectively amplify ventilator-associated adverse outcomes. We advocate for individualized clinical evaluation integrating COPD status, serum creatinine, and anion gap to guide tailored respiratory support decisions in sepsis management. The persistent associations at 90-day follow-up underscore the durability of these relationships. Future prospective multicenter studies should examine how these clinical and metabolic parameters modify ventilator-associated outcomes and assess whether tailored ventilation protocols benefit patient subgroups with specific physiological alterations, potentially advancing personalized sepsis management.

## Data Availability

The raw data supporting the conclusions of this article will be made available by the authors upon reasonable request. Due to ethical restrictions and patient privacy regulations in China, individual-level clinical data cannot be publicly deposited. Requests to access the datasets should be directed to the corresponding author (Yan Xue, gbx99@fjmu.edu.cn).

## Glossary

AG: anion gap
ALB: albumin
APACHE II: Acute Physiology and Chronic Health Evaluation II
ARDS: acute respiratory distress syndrome
ARDS Net: ARDS Network
BMI: body mass index
BUN: blood urea nitrogen
CHF: congestive heart failure
CI: confidence interval
CKD: chronic kidney disease
CLD: chronic liver disease
COPD: chronic obstructive pulmonary disease
CRP: C-reactive protein
DBIL: direct bilirubin
DM: diabetes mellitus
FiO₂: fraction of inspired oxygen
HCO₃^−^: bicarbonate
HR: heart rate
HTN: hypertension
ICU: intensive care unit
INR: international normalized ratio
LDH: lactate dehydrogenase
MAP: mean arterial pressure
MV: mechanical ventilation
NIV: non-invasive ventilation
OR: odds ratio
PCT: procalcitonin
PEEP: positive end-expiratory pressure
PLT: platelet count
SCr: serum creatinine
SpO₂: oxygen saturation
VALI: ventilator-associated lung injury
VIF: variance inflation factor

## References

[1] Rudd KE Johnson SC Agesa KM Shackelford KA Tsoi D Kievlan DR . Global, regional, and national sepsis incidence and mortality, 1990-2017: analysis for the global burden of disease study. Lancet. (2020) 395:200–11. doi: 10.1016/S0140-6736(19)32989-7, 31954465 PMC6970225

[2] Singer M Deutschman CS Seymour CW Shankar-Hari M Annane D Bauer M . The third international consensus definitions for sepsis and septic shock (Sepsis-3). JAMA. (2016) 315:801–10. doi: 10.1001/jama.2016.0287, 26903338 PMC4968574

[3] Feng Z Wang L Yang J Li T Liao X Kang Y . Sepsis: the evolution of molecular pathogenesis concepts and clinical management. MedComm. (2020) 6:e70109. doi: 10.1002/mco2.70109 PMC11847631 39991626

[4] World Health Organization. Sepsis [Internet]. Geneva: World Health Organization (2024).

[5] Xie J Wang H Kang Y Zhou L Liu Z Qin B . The epidemiology of sepsis in Chinese ICUs: a national cross-sectional survey. Crit Care Med. (2020) 48:e209–18. doi: 10.1097/CCM.0000000000004155, 31804299

[6] Weng L Xu Y Yin P Wang Y Chen Y Liu W . National incidence and mortality of hospitalized sepsis in China. Crit Care. (2023) 27:84. doi: 10.1186/s13054-023-04385-x, 36870989 PMC9985297

[7] Liu YC Yao Y Yu MM Gao YL Qi AL Jiang TY . Frequency and mortality of sepsis and septic shock in China: a systematic review and meta-analysis. BMC Infect Dis. (2022) 22:564. doi: 10.1186/s12879-022-07543-8, 35729526 PMC9210671

[8] Papazian L Aubron C Brochard L Chiche JD Combes A Dreyfuss D . Formal guidelines: management of acute respiratory distress syndrome. Ann Intensive Care. (2019) 9:69. doi: 10.1186/s13613-019-0540-9, 31197492 PMC6565761

[9] Slutsky AS Ranieri VM. Ventilator-induced lung injury. N Engl J Med. (2013) 369:2126–36. doi: 10.1056/NEJMra1208707 24283226

[10] Liu N Ren J Yu L Xie J. Mechanical ventilation associated with worse survival in septic patients: a retrospective analysis of MIMIC-III. J Emerg Crit Care Med. (2020) 4:14. doi: 10.21037/jeccm.2020.01.01

[11] Bellani G Laffey JG Pham T Fan E Brochard L Esteban A . Epidemiology, patterns of care, and mortality for patients with acute respiratory distress syndrome in intensive care units in 50 countries. JAMA. (2016) 315:788–800. doi: 10.1001/jama.2016.0291, 26903337

[12] Cecconi M Evans L Levy M Rhodes A. Sepsis and septic shock. Lancet. (2018) 392:75–87. doi: 10.1016/S0140-6736(18)30696-2 29937192

[13] Chen Y Lu L Li X Liu B Zhang Y Zheng Y . Association between chronic obstructive pulmonary disease and 28-day mortality in patients with sepsis: a retrospective study based on the MIMIC-III database. BMC Pulm Med. (2023) 23:435. doi: 10.1186/s12890-023-02729-5, 37946194 PMC10633936

[14] Zhang L Ye S Hu J Huang Z Lin X Lin Y . Group-based trajectory modeling of anion gap and mortality in patients with sepsis: a retrospective analysis of the MIMIC-IV database. Eur J Med Res. (2025) 30:879. doi: 10.1186/s40001-025-03146-6, 41013599 PMC12465563

[15] Li Q Li G Li D Chen Y Zhou F. Early and minimal changes in serum creatinine can predict prognosis in elderly patients receiving invasive mechanical ventilation: a retrospective observational study. J Intensive Med. (2024) 4:368–75. doi: 10.1016/j.jointm.2023.10.003, 39035610 PMC11258507

[16] Lou Z Zeng F Huang W Xiao L Zou K Zhou H. Association between the anion-gap and 28-day mortality in critically ill adult patients with sepsis: a retrospective cohort study. Medicine (Baltimore). (2024) 103:e39029. doi: 10.1097/MD.0000000000039029, 39058855 PMC11272324

[17] Thongprayoon C Cheungpasitporn W Kashani K. Serum creatinine level, a surrogate of muscle mass, predicts mortality in critically ill patients. J Thorac Dis. (2016) 8:E305–11. doi: 10.21037/jtd.2016.03.62, 27162688 PMC4842835

[18] Demoule A Brochard L Dres M Heunks L Jubran A Laghi F . How to ventilate obstructive and asthmatic patients. Intensive Care Med. (2020) 46:2436–49. doi: 10.1007/s00134-020-06291-0, 33169215 PMC7652057

[19] Rodríguez-Villar S Kraut JA Arévalo-Serrano J Sakka SG Harris C Awad I . Systemic acidemia impairs cardiac function in critically ill patients. EClinicalMedicine. (2021) 37:100956. doi: 10.1016/j.eclinm.2021.100956, 34258569 PMC8255172

[20] White KC Bellomo R Laupland KB Gatton ML Ostermann M McIlroy P . Predicting a strongly positive fluid balance in critically ill patients with acute kidney injury: a multicentre, international study. J Crit Care. (2025) 87:155016. doi: 10.1016/j.jcrc.2025.155016, 39855144

[21] Messmer AS Zingg C Müller M Gerber JL Schefold JC Pfortmueller CA. Fluid overload and mortality in adult critical care patients-a systematic review and meta-analysis of observational studies. Crit Care Med. (2020) 48:1862–70. doi: 10.1097/CCM.0000000000004617, 33009098

[22] Lu Y Zhang J Zhang W Shi H Wang K Li Z . Impact of initial ventilation strategies on in-hospital mortality in sepsis patients: insights from the MIMIC-IV database. BMC Pulm Med. (2025) 25:147. doi: 10.1186/s12890-025-03610-3, 40170136 PMC11959717

[23] Jivraj NK Hill AD Shieh MS Hua M Gershengorn HB Ferrando-Vivas P . Use of mechanical ventilation across 3 countries. JAMA Intern Med. (2023) 183:824–31. doi: 10.1001/jamainternmed.2023.2371, 37358834 PMC10294017

[24] Kraut JA Madias NE. Serum anion gap: its uses and limitations in clinical medicine. Clin J Am Soc Nephrol. (2007) 2:162–74. doi: 10.2215/CJN.03020906 17699401

[25] Brower RG Matthay MA Morris A Schoenfeld D Thompson BT Wheeler A. Ventilation with lower tidal volumes as compared with traditional tidal volumes for acute lung injury and the acute respiratory distress syndrome. N Engl J Med. (2000) 342:1301–8. doi: 10.1056/NEJM200005043421801, 10793162

[26] Evans L Rhodes A Alhazzani W Antonelli M Coopersmith CM French C . Surviving sepsis campaign: international guidelines for management of sepsis and septic shock 2021. Crit Care Med. (2021) 49:e1063–143. doi: 10.1097/CCM.0000000000005337, 34605781

[27] Gilbert DN Chambers HF Saag MS Pavia AT Black HW Boucher D. The Sanford Guide to Antimicrobial Therapy. 53rd ed. Beijing: Peking Union Medical College Press (2024).

[28] Kim G Oh DK Lee SY Park MH Lim CM. Impact of the timing of invasive mechanical ventilation in patients with sepsis: a multicenter cohort study. Crit Care. (2024) 28:297. doi: 10.1186/s13054-024-05064-1, 39252133 PMC11385489

[29] Zhang L Zhang H Huang Z Ye S Sun X Lin R . Relationship between time-weighted average anion gap and mortality in septic shock patients: a retrospective analysis of the MIMIC-IV database. BMC Infect Dis. (2025) 25:961. doi: 10.1186/s12879-025-11329-z, 40739194 PMC12312503

[30] Koehler P Bassetti M Chakrabarti A Chen SCA Colombo AL Hoenigl M . Defining and managing COVID-19-associated pulmonary aspergillosis: the 2020 ECMM/ISHAM consensus criteria for research and clinical guidance. Lancet Infect Dis. (2021) 21:e149–62. doi: 10.1016/S1473-3099(20)30847-1, 33333012 PMC7833078

[31] Curley GF Laffey JG Zhang H Slutsky AS. Biotrauma and ventilator-induced lung injury: clinical implications. Chest. (2016) 150:1109–17. doi: 10.1016/j.chest.2016.07.019 27477213

[32] Meyer NJ Gattinoni L Calfee CS. Acute respiratory distress syndrome. Lancet. (2021) 398:622–37. doi: 10.1016/S0140-6736(21)00439-6, 34217425 PMC8248927

[33] Matthay MA Zemans RL Zimmerman GA Arabi YM Beitler JR Mercat A . Acute respiratory distress syndrome. Nat Rev Dis Primers. (2019) 5:18. doi: 10.1038/s41572-019-0069-0, 30872586 PMC6709677

[34] Kraut JA Madias NE. Metabolic acidosis: pathophysiology, diagnosis and management. Nat Rev Nephrol. (2010) 6:274–85. doi: 10.1038/nrneph.2010.33 20308999

[35] Garcia SI Smischney NJ Sandefur BJ D'Andria Ursoleo J Kelm DJ Wieruszewski PM. Peri-intubation cardiovascular collapse during emergency airway management. Pulm Ther. (2025) 11:569–85. doi: 10.1007/s41030-025-00326-x, 41094343 PMC12623605

[36] Suga M Nishimura T Ochi T Hongo T Yumoto T Nakao A . Association between metabolic acidosis and post-intubation hypotension in airway management performed in the emergency department. Heliyon. (2024) 10:e40224. doi: 10.1016/j.heliyon.2024.e40224, 39660193 PMC11629204

[37] Lipman J Lewis RE. The long walk to a short half-life: the discovery of augmented renal clearance and its impact on antibiotic dosing. J Antimicrob Chemother. (2025) 80:3367–74. doi: 10.1093/jac/dkaf378, 41077962 PMC12670167

[38] Roberts JA Paul SK Akova M Bassetti M De Waele JJ Dimopoulos G . DALI: defining antibiotic levels in intensive care unit patients: are current β-lactam antibiotic doses sufficient for critically ill patients? Clin Infect Dis. (2014) 58:1072–83. doi: 10.1093/cid/ciu027, 24429437

[39] Thongprayoon C Cheungpasitporn W Chewcharat A Mao MA Thirunavukkarasu S Kashani KB. The association of low admission serum creatinine with the risk of respiratory failure requiring mechanical ventilation: a retrospective cohort study. Sci Rep. (2019) 9:18743. doi: 10.1038/s41598-019-55362-w, 31822769 PMC6904463

[40] Sklar MC Dres M Fan E Rubenfeld GD Scales DC Herridge MS . Association of low baseline diaphragm muscle mass with prolonged mechanical ventilation and mortality among critically ill adults. JAMA Netw Open. (2020) 3:e1921520. doi: 10.1001/jamanetworkopen.2019.21520, 32074293 PMC12124489

[41] Zhu J Zhang Z Lei Y Ouyang Z Kutty S Liu Q . The prediction value of serum anion gap for short-term mortality in pulmonary hypertension patients with sepsis: a retrospective cohort study. Front Med (Lausanne). (2024) 11:1499677. doi: 10.3389/fmed.2024.1499677 39839613 PMC11748302

[42] Li R Jin X Ren J Deng G Li J Gao Y . Relationship of admission serum anion gap and prognosis of critically ill patients: a large multicenter cohort study. Dis Markers. (2022) 2022:1–10. doi: 10.1155/2022/5926049 PMC9771639 36569219

[43] Rubens M Appunni S Saxena A Ramamoorthy V Mathew C Khosla AA . Association of serum anion gap and mortality in critically ill patients receiving mechanical circulatory support. Am J Crit Care. (2025) 34:419–28. doi: 10.4037/ajcc2025708, 41173647

[44] Choudhary T Upadhyaya P Davis CM Yang P Tallowin S Lisboa FA . Derivation and validation of generalized sepsis-induced acute respiratory failure phenotypes among critically ill patients: a retrospective study. Crit Care. (2024) 28:321. doi: 10.1186/s13054-024-05061-4, 39354616 PMC11445942

[45] Evans L Rhodes A Alhazzani W Antonelli M Coopersmith CM French C . Surviving sepsis campaign: international guidelines for management of sepsis and septic shock 2021. Intensive Care Med. (2021) 47:1181–247. doi: 10.1007/s00134-021-06506-y, 34599691 PMC8486643

[46] Baptista L Moura I Silva CM Baptista JP. What is new in augmented renal clearance in septic patients? Curr Infect Dis Rep. (2023) 25:255–72. doi: 10.1007/s11908-023-00816-6

[47] Goligher EC Dres M Patel BK Sahetya SK Beitler JR Telias I . Lung- and diaphragm-protective ventilation. Am J Respir Crit Care Med. (2020) 202:950–61. doi: 10.1164/rccm.202003-0655CP, 32516052 PMC7710325

[48] Mein SA Ferrera MC. Management of asthma and COPD exacerbations in adults in the ICU. CHEST Crit Care. (2025) 3:100107. doi: 10.1016/j.chstcc.2024.100107 40330435 PMC12054689

