# Supplementary material for: Effect modification by chronic obstructive pulmonary disease, anion gap, and serum creatinine on the association between invasive mechanical ventilation and 28-day mortality in intensive care unit sepsis patients: a retrospective cohort study
Source: Front Med (Lausanne). 2026 May 27;13:1758229. doi: 10.3389/fmed.2026.1758229 (PMC13252744; doi:10.3389/fmed.2026.1758229)
Supplement: Supplementary file 1 [file Data_Sheet_1.pdf]

Supplemental Tables

Supplemental Table S1. Stratified Analysis of MV-28-Day Mortality Association

Stratification Variable	*Stratum*	*n*	*OR (95% CI)*	*p*	*Pinteraction*
**AGE**	Low	232	2.67 (1.44, 4.95)	0.002	0.803
	Medium	217	4.57 (2.23, 9.36)	<0.001	
	High	224	2.19 (1.13, 4.24)	0.020	
**SEX**	Male	422	2.60 (1.61, 4.20)	<0.001	0.303
	Female	251	3.91 (2.12, 7.22)	<0.001	
**BMI**	Low	236	3.09 (1.63, 5.85)	<0.001	0.954
	Medium	216	3.47 (1.80, 6.67)	<0.001	
	High	221	2.55 (1.30, 4.99)	0.006	
**DM**	No	438	2.71 (1.68, 4.38)	<0.001	0.433
	Yes	235	3.70 (2.01, 6.82)	<0.001	
**HTN**	No	390	4.17 (2.48, 7.04)	<0.001	0.069
	Yes	283	2.06 (1.18, 3.59)	0.011	
**APACHE II**	Low	242	3.42 (1.96, 5.97)	<0.001	0.132
	Medium	225	2.45 (1.16, 5.18)	0.019	
	High	206	1.11 (0.43, 2.89)	0.830	
**HR**	Low	230	2.38 (1.30, 4.35)	0.005	0.785
	Medium	224	5.71 (2.69, 12.09)	<0.001	
	High	219	1.99 (1.01, 3.92)	0.048	
**MAP**	Low	227	6.12 (3.09, 12.14)	<0.001	0.097
	Medium	226	2.17 (1.15, 4.10)	0.017	
	High	220	2.08 (1.07, 4.04)	0.032	
**PLT**	Low	224	4.25 (2.30, 7.85)	<0.001	0.327
	Medium	224	2.14 (1.12, 4.09)	0.022	
	High	225	3.69 (1.74, 7.85)	<0.001	

Stratification Variable	*Stratum*	*n*	*OR (95% CI)*	*p*	*Pinteraction*
**INR**	Low	236	3.48 (1.82, 6.66)	<0.001	0.192
	Medium	213	2.36 (1.25, 4.46)	0.008	
	High	224	3.18 (1.59, 6.36)	0.001	
**ALB**	Low	225	2.08 (1.01, 4.29)	0.048	0.380
	Medium	227	4.00 (2.13, 7.49)	<0.001	
	High	221	2.95 (1.56, 5.57)	<0.001	
**DBIL**	Low	224	2.39 (1.27, 4.50)	0.007	0.722
	Medium	224	2.61 (1.35, 5.03)	0.004	
	High	225	4.57 (2.33, 8.95)	<0.001	
**HCO_3^-**	Low	231	4.45 (2.38, 8.30)	<0.001	0.529
	Medium	219	2.97 (1.57, 5.60)	<0.001	
	High	223	2.26 (1.09, 4.70)	0.028	
**AG**	Low	225	2.40 (1.19, 4.82)	0.014	0.017
	Medium	230	2.05 (1.13, 3.72)	0.018	
	High	218	6.62 (3.28, 13.38)	<0.001	
**SCr**	Low	227	3.73 (1.85, 7.54)	<0.001	0.020
	Medium	223	2.85 (1.50, 5.44)	0.001	
	High	223	2.69 (1.41, 5.12)	0.003	
**BUN**	Low	226	4.88 (2.32, 10.27)	<0.001	0.114
	Medium	223	3.67 (1.75, 7.67)	<0.001	
	High	224	2.15 (1.18, 3.94)	0.013	
**LDH**	Low	225	2.68 (1.32, 5.42)	0.006	0.218
	Medium	222	2.48 (1.36, 4.53)	0.003	
	High	226	3.81 (1.85, 7.84)	<0.001	
**PCT**	Low	219	1.85 (0.99, 3.49)	0.055	0.972
	Medium	229	5.47 (2.65, 11.30)	<0.001	
	High	225	2.96 (1.56, 5.62)	<0.001	
**CRP**	Low	225	2.31 (1.20, 4.44)	0.013	0.950

Stratification Variable	Stratum	n	OR (95% CI)	p	Pinteraction
	Medium	329	3.98 (2.28, 6.96)	<0.001	
	High	119	2.44 (1.07, 5.60)	0.035	
**COPD**	No	630	3.23 (2.19, 4.78)	<0.001	0.043
	Yes	43	0.38 (0.04, 3.77)	0.411	
**CHF**	No	556	3.04 (1.99, 4.66)	<0.001	0.810
	Yes	117	3.41 (1.48, 7.87)	0.004	
**CKD**	No	481	3.39 (2.13, 5.38)	<0.001	0.457
	Yes	192	2.48 (1.27, 4.87)	0.008	
**CLD**	No	666	3.05 (2.08, 4.46)	<0.001	—
	Yes	7	N/A	N/A	
**CVA**	No	572	3.05 (2.03, 4.57)	<0.001	0.902
	Yes	101	2.84 (1.02, 7.89)	0.045	

Abbreviations: OR, odds ratio; CI, confidence interval; MV, mechanical ventilation; AGE, age; SEX, sex; BMI, body mass index; DM, diabetes mellitus; HTN, hypertension; APACHE II, Acute Physiology and Chronic Health Evaluation II; HR, heart rate; MAP, mean arterial pressure; PLT, platelet count; INR, international normalized ratio; ALB, albumin; DBIL, direct bilirubin; HCO_3^-, bicarbonate; AG, anion gap; SCr, serum creatinine; BUN, blood urea nitrogen; LDH, lactate dehydrogenase; PCT, procalcitonin; CRP, C-reactive protein; COPD, chronic obstructive pulmonary disease; CHF, congestive heart failure; CKD, chronic kidney disease; CLD, chronic liver disease; CVA, cerebrovascular accident.

§The model failed because of the small sample size.

Pinteraction values indicate whether the effect modification by stratification variable is statistically significant.

Statistical significance was set at $p<0.05$ (two-sided).

Supplemental Table S2. Stratified Analysis of MV-90-Day Mortality Association

Stratification Variable	*Stratum*	*n*	*OR (95% CI)*	*p*	*Pinteraction*
**AGE**	Low	232	2.80 (1.51, 5.20)	0.001	0.698
	Medium	217	4.46 (2.18, 9.13)	<0.001	
	High	224	2.47 (1.27, 4.79)	0.007	
**SEX**	Male	422	2.68 (1.66, 4.31)	<0.001	0.251
	Female	251	4.22 (2.27, 7.87)	<0.001	
**BMI**	Low	235	2.95 (1.61, 5.41)	<0.001	0.854
	Medium	219	5.10 (2.46, 10.55)	<0.001	
	High	219	2.23 (1.14, 4.37)	0.020	
**DM**	No	438	2.88 (1.77, 4.68)	<0.001	0.442
	Yes	235	3.90 (2.13, 7.16)	<0.001	
**HTN**	No	390	4.29 (2.54, 7.24)	<0.001	0.091
	Yes	283	2.23 (1.28, 3.88)	0.005	
**APACHE II**	Low	233	3.61 (2.07, 6.30)	<0.001	0.271
	Medium	228	2.85 (1.25, 6.50)	0.013	
	High	212	1.65 (0.70, 3.87)	0.251	
**HR**	Low	230	2.28 (1.25, 4.14)	0.007	0.534
	Medium	224	6.13 (2.89, 13.01)	<0.001	
	High	219	2.30 (1.16, 4.55)	0.017	
**MAP**	Low	227	5.82 (2.94, 11.53)	<0.001	0.110
	Medium	227	2.55 (1.34, 4.85)	0.004	
	High	219	2.11 (1.09, 4.07)	0.026	
**PLT**	Low	224	4.25 (2.30, 7.85)	<0.001	0.271
	Medium	225	2.71 (1.41, 5.24)	0.003	
	High	224	3.23 (1.55, 6.73)	0.002	
**INR**	Low	236	3.35 (1.77, 6.35)	<0.001	0.212

Stratification Variable	*Stratum*	*n*	*OR (95% CI)*	*p*	*Pinteraction*
	Medium	213	2.69 (1.42, 5.08)	0.002	
	High	224	3.45 (1.71, 6.96)	<0.001	
**ALB**	Low	225	2.15 (1.05, 4.41)	0.037	0.365
	Medium	226	4.46 (2.35, 8.45)	<0.001	
	High	222	3.05 (1.63, 5.72)	<0.001	
**DBIL**	Low	226	2.76 (1.46, 5.20)	0.002	0.574
	Medium	224	2.39 (1.25, 4.57)	0.008	
	High	223	5.06 (2.55, 10.03)	<0.001	
**HCO_3^-**	Low	232	4.98 (2.65, 9.36)	<0.001	0.387
	Medium	219	2.97 (1.57, 5.60)	<0.001	
	High	222	2.23 (1.09, 4.57)	0.028	
**AG**	Low	225	2.51 (1.25, 5.04)	0.010	0.013
	Medium	230	2.15 (1.19, 3.91)	0.012	
	High	218	7.03 (3.47, 14.23)	<0.001	
**SCr**	Low	227	3.65 (1.81, 7.36)	<0.001	0.141
	Medium	223	2.76 (1.46, 5.22)	0.002	
	High	223	3.32 (1.73, 6.38)	<0.001	
**BUN**	Low	226	4.25 (2.06, 8.73)	<0.001	0.602
	Medium	223	3.75 (1.79, 7.84)	<0.001	
	High	224	2.73 (1.48, 5.02)	0.001	
**LDH**	Low	225	3.22 (1.56, 6.63)	0.002	0.270
	Medium	222	2.48 (1.36, 4.53)	0.003	
	High	226	3.69 (1.80, 7.59)	<0.001	
**PCT**	Low	224	1.99 (1.06, 3.73)	0.032	0.804
	Medium	219	5.53 (2.61, 11.71)	<0.001	
	High	230	3.27 (1.72, 6.21)	<0.001	
**CRP**	Low	225	2.59 (1.34, 4.99)	0.005	0.872
	Medium	329	4.05 (2.32, 7.08)	<0.001	

Stratification Variable	Stratum	n	OR (95% CI)	p	Pinteraction
	High	119	2.44 (1.07, 5.60)	0.035	
**COPD**	No	630	3.41 (2.31, 5.03)	<0.001	0.038
	Yes	43	0.38 (0.04, 3.77)	0.411	
**CHF**	No	556	3.19 (2.08, 4.90)	<0.001	0.648
	Yes	117	3.97 (1.72, 9.19)	0.001	
**CKD**	No	481	3.24 (2.05, 5.13)	<0.001	0.992
	Yes	192	3.26 (1.65, 6.44)	<0.001	
**CLD**	No	666	3.20 (2.19, 4.68)	<0.001	—
	Yes	7	N/A	N/A	
**CVA**	No	572	3.06 (2.04, 4.59)	<0.001	0.651
	Yes	101	3.97 (1.38, 11.42)	0.011	

Abbreviations: OR, odds ratio; CI, confidence interval; MV, mechanical ventilation; AGE, age; SEX, sex; BMI, body mass index; DM, diabetes mellitus; HTN, hypertension; APACHE II, Acute Physiology and Chronic Health Evaluation II; HR, heart rate; MAP, mean arterial pressure; PLT, platelet count; INR, international normalized ratio; ALB, albumin; DBIL, direct bilirubin; HCO_3^-, bicarbonate; AG, anion gap; SCr, serum creatinine; BUN, blood urea nitrogen; LDH, lactate dehydrogenase; PCT, procalcitonin; CRP, C-reactive protein; COPD, chronic obstructive pulmonary disease; CHF, congestive heart failure; CKD, chronic kidney disease; CLD, chronic liver disease; CVA, cerebrovascular accident.

§The model failed because of the small sample size.

Pinteraction values indicate whether the effect modification by stratification variable is statistically significant.

Statistical significance was set at *p*<0.05 (two-sided).

Supplemental Table S3. Missing Data Analysis for Primary Outcome (28-Day Mortality)

Variable	Complete Cases (n)	Missing Cases (n)	Missing Rate (%)	Handling Method
**Demographics**				
Age	673	0	0.00	missForest
Sex	673	0	0.00	missForest
BMI	544	129	19.17	missForest
**Disease Severity**				
APACHE II score	598	75	11.14	missForest
**Hemodynamic Parameters**				
Heart rate	673	0	0.00	missForest
Mean arterial pressure	672	1	0.15	missForest
**Biomarkers and Laboratory**				
Procalcitonin	620	53	7.88	missForest
C-reactive protein	673	0	0.00	missForest
**Renal Function**				
Serum Creatinine	673	0	0.00	missForest
Blood urea nitrogen	673	0	0.00	missForest
**Metabolic Parameters**				
Anion gap	673	0	0.00	missForest
Bicarbonate	672	1	0.15	missForest
**Hepatic Function and Nutrition**				
Albumin	669	4	0.59	missForest
Direct bilirubin	669	4	0.59	missForest
Lactate dehydrogenase	668	5	0.74	missForest
**Hematologic Parameters**				
Platelet count	672	1	0.15	missForest
International normalized ratio	672	1	0.15	missForest

Variable	Complete Cases (n)	Missing Cases (n)	Missing Rate (%)	Handling Method
**Comorbidities**				
Diabetes mellitus	673	0	0.00	missForest
Hypertension	673	0	0.00	missForest
Chronic obstructive pulmonary disease	673	0	0.00	missForest
Congestive heart failure	673	0	0.00	missForest
Chronic kidney disease	673	0	0.00	missForest
Chronic liver disease	673	0	0.00	missForest
Cerebrovascular accident	673	0	0.00	missForest
**Treatment Variables**				
Antibiotic therapy	673	0	0.00	missForest

Variables with missing rate ≥40% were excluded from analysis. Since multiple variables (BMI, APACHE II score, Procalcitonin) had missing rates >5%, all covariates were imputed using the missForest algorithm for methodological consistency, regardless of their individual missing rates. Final sample size after processing: 673 patients. All statistical analyses were performed using Python version 3.10.12.

Supplemental Table S4. Comparison of Original and Imputed Data for Primary Analysis (28-Day Mortality)

Variable	Original Data	Imp 1	Imp 2	Imp 3	Imp 4	Imp 5	Test	*p*-value
**Demographics**								
Age (years)	70.25 ± 14.17 (n=673)	70.25 ± 14.17 (n=673)	70.25 ± 14.17 (n=673)	70.25 ± 14.17 (n=673)	70.25 ± 14.17 (n=673)	70.25 ± 14.17 (n=673)	N/A†	—
**Sex**								
Male	422 (62.7%)	422 (62.7%)	422 (62.7%)	422 (62.7%)	422 (62.7%)	422 (62.7%)	N/A†	—
Female	251 (37.3%)	251 (37.3%)	251 (37.3%)	251 (37.3%)	251 (37.3%)	251 (37.3%)		
BMI (kg/m²)*	22.41 ± 4.05 (n=544)	22.30 ± 4.16 (n=673)	22.48 ± 4.09 (n=673)	22.46 ± 4.09 (n=673)	22.35 ± 4.08 (n=673)	22.40 ± 4.09 (n=673)	ANOVA	0.970
**Disease Severity**								
APACHE II score*	22.60 ± 7.07 (n=598)	22.40 ± 7.13 (n=673)	22.43 ± 7.17 (n=673)	22.54 ± 7.25 (n=673)	22.45 ± 7.14 (n=673)	22.74 ± 7.11 (n=673)	ANOVA	0.953
**Hemodynamic Parameters**								
Heart rate (bpm)	108.23 ± 23.19 (n=673)	108.23 ± 23.19 (n=673)	108.23 ± 23.19 (n=673)	108.23 ± 23.19 (n=673)	108.23 ± 23.19 (n=673)	108.23 ± 23.19 (n=673)	N/A†	—
MAP (mmHg)*	85.54 ± 18.87 (n=672)	85.56 ± 18.86 (n=673)	85.56 ± 18.86 (n=673)	85.56 ± 18.86 (n=673)	85.53 ± 18.86 (n=673)	85.52 ± 18.86 (n=673)	ANOVA	1.000
**Biomarkers and Laboratory**								
Procalcitonin (ng/mL)*	61.53 ± 67.31 (n=620)	61.25 ± 65.02 (n=673)	61.32 ± 65.03 (n=673)	61.23 ± 65.06 (n=673)	61.24 ± 65.02 (n=673)	61.31 ± 65.03 (n=673)	KW	0.997
CRP (mg/L)	144.99 ± 86.50 (n=673)	144.99 ± 86.50 (n=673)	144.99 ± 86.50 (n=673)	144.99 ± 86.50 (n=673)	144.99 ± 86.50 (n=673)	144.99 ± 86.50 (n=673)	N/A†	—
**Renal Function**								
Serum Creatinine (µmol/L)	180.99 ± 152.74 (n=673)	180.99 ± 152.74 (n=673)	180.99 ± 152.74 (n=673)	180.99 ± 152.74 (n=673)	180.99 ± 152.74 (n=673)	180.99 ± 152.74 (n=673)	N/A†	—
BUN (mmol/L)	14.69 ± 10.21 (n=673)	14.69 ± 10.21 (n=673)	14.69 ± 10.21 (n=673)	14.69 ± 10.21 (n=673)	14.69 ± 10.21 (n=673)	14.69 ± 10.21 (n=673)	N/A†	—
**Metabolic**								

Variable	Original Data	Imp 1	Imp 2	Imp 3	Imp 4	Imp 5	Test	*p*-value
**Parameters**								
Anion gap (mmol/L)	15.24 ± 5.75 (n=673)	15.24 ± 5.75 (n=673)	15.24 ± 5.75 (n=673)	15.24 ± 5.75 (n=673)	15.24 ± 5.75 (n=673)	15.24 ± 5.75 (n=673)	N/A†	—
Bicarbonate (mmol/L)*	19.20 ± 5.21 (n=672)	19.20 ± 5.20 (n=673)	19.20 ± 5.20 (n=673)	19.20 ± 5.20 (n=673)	19.19 ± 5.21 (n=673)	19.19 ± 5.21 (n=673)	ANOVA	1.000
**Hepatic Function and Nutrition**								
Albumin (g/L)*	25.64 ± 6.38 (n=669)	25.63 ± 6.37 (n=673)	25.61 ± 6.38 (n=673)	25.64 ± 6.37 (n=673)	25.65 ± 6.41 (n=673)	25.62 ± 6.38 (n=673)	ANOVA	1.000
Direct bilirubin (μmol/L)*	18.71 ± 30.87 (n=669)	18.70 ± 30.78 (n=673)	18.63 ± 30.79 (n=673)	18.72 ± 30.81 (n=673)	18.77 ± 30.90 (n=673)	18.63 ± 30.79 (n=673)	ANOVA	1.000
LDH (U/L)*	644.28 ± 1774.42 (n=668)	647.94 ± 1769.14 (n=673)	647.60 ± 1769.20 (n=673)	646.91 ± 1768.85 (n=673)	646.64 ± 1768.65 (n=673)	647.84 ± 1769.64 (n=673)	KW	1.000
**Hematologic Parameters**								
Platelet count ($\times 10^9$/L)*	160.84 ± 104.92 (n=672)	160.96 ± 104.89 (n=673)	160.69 ± 104.92 (n=673)	160.95 ± 104.88 (n=673)	160.88 ± 104.85 (n=673)	160.69 ± 104.92 (n=673)	ANOVA	1.000
INR*	1.46 ± 0.48 (n=672)	1.46 ± 0.48 (n=673)	1.46 ± 0.48 (n=673)	1.46 ± 0.48 (n=673)	1.46 ± 0.48 (n=673)	1.46 ± 0.48 (n=673)	ANOVA	1.000
**Comorbidities**								
**Diabetes mellitus**								
No	438 (65.1%)	438 (65.1%)	438 (65.1%)	438 (65.1%)	438 (65.1%)	438 (65.1%)	N/A†	—
Yes	235 (34.9%)	235 (34.9%)	235 (34.9%)	235 (34.9%)	235 (34.9%)	235 (34.9%)		
**Hypertension**								
No	390 (57.9%)	390 (57.9%)	390 (57.9%)	390 (57.9%)	390 (57.9%)	390 (57.9%)	N/A†	—
Yes	283 (42.1%)	283 (42.1%)	283 (42.1%)	283 (42.1%)	283 (42.1%)	283 (42.1%)		
**Chronic obstructive pulmonary disease**								
No	630 (93.6%)	630 (93.6%)	630 (93.6%)	630 (93.6%)	630 (93.6%)	630 (93.6%)	N/A†	—
Yes	43 (6.4%)	43 (6.4%)	43 (6.4%)	43 (6.4%)	43 (6.4%)	43 (6.4%)		
**Congestive heart failure**								
No	556 (82.6%)	556 (82.6%)	556 (82.6%)	556 (82.6%)	556 (82.6%)	556 (82.6%)	N/A†	—
Yes	117 (17.4%)	117 (17.4%)	117 (17.4%)	117 (17.4%)	117 (17.4%)	117 (17.4%)		

Variable	Original Data	Imp 1	Imp 2	Imp 3	Imp 4	Imp 5	Test	*p*-value
**Chronic kidney disease**								
No	481 (71.5%)	481 (71.5%)	481 (71.5%)	481 (71.5%)	481 (71.5%)	481 (71.5%)	N/A†	—
Yes	192 (28.5%)	192 (28.5%)	192 (28.5%)	192 (28.5%)	192 (28.5%)	192 (28.5%)		
**Chronic liver disease**								
No	666 (99.0%)	666 (99.0%)	666 (99.0%)	666 (99.0%)	666 (99.0%)	666 (99.0%)	N/A†	—
Yes	7 (1.0%)	7 (1.0%)	7 (1.0%)	7 (1.0%)	7 (1.0%)	7 (1.0%)		
**Cerebrovascular accident**								
No	572 (85.0%)	572 (85.0%)	572 (85.0%)	572 (85.0%)	572 (85.0%)	572 (85.0%)	N/A†	—
Yes	101 (15.0%)	101 (15.0%)	101 (15.0%)	101 (15.0%)	101 (15.0%)	101 (15.0%)		
**Treatment Variables**								
**Antibiotic therapy**								
No antibacterial	19 (2.8%)	19 (2.8%)	19 (2.8%)	19 (2.8%)	19 (2.8%)	19 (2.8%)	N/A†	—
Narrow-spectrum	39 (5.8%)	39 (5.8%)	39 (5.8%)	39 (5.8%)	39 (5.8%)	39 (5.8%)		
Broad-spectrum	469 (69.7%)	469 (69.7%)	469 (69.7%)	469 (69.7%)	469 (69.7%)	469 (69.7%)		
Combination therapy	146 (21.7%)	146 (21.7%)	146 (21.7%)	146 (21.7%)	146 (21.7%)	146 (21.7%)		

Values for continuous variables are presented as mean ± standard deviation. Categorical variables are presented as counts (percentage). *Variables with missing values requiring imputation. †Not applicable (no statistical comparison performed for variables with no missing values). Statistical comparisons were performed using one-way ANOVA for normally distributed variables or Kruskal-Wallis test (KW) for non-normally distributed variables. All statistical analyses were performed using Python version 3.10.12.

Supplemental Table S5. Multiple Imputation Analysis Results for Mechanical Ventilation and 28-Day Mortality

Analysis	Unadjusted OR (95% CI)	*p* value	Adjusted OR (95% CI)*	*p* value
Imputation 1	3.04 (2.09, 4.43)	<0.001	2.60 (1.67, 4.05)	<0.001
Imputation 2	3.04 (2.09, 4.43)	<0.001	2.54 (1.64, 3.96)	<0.001
Imputation 3	3.04 (2.09, 4.43)	<0.001	2.63 (1.69, 4.10)	<0.001
Imputation 4	3.04 (2.09, 4.43)	<0.001	2.76 (1.76, 4.31)	<0.001
Imputation 5	3.04 (2.09, 4.43)	<0.001	2.57 (1.65, 4.02)	<0.001
Pooled Result†	3.04 (2.09, 4.43)	<0.001	2.62 (1.67, 4.11)	<0.001

Results from each individual imputation dataset and pooled analysis using Rubin's rules.

*Adjusted for age, sex, APACHE II score, comorbidities (diabetes mellitus, hypertension, chronic obstructive pulmonary disease, congestive heart failure, chronic kidney disease, chronic liver disease, cerebrovascular accident), and laboratory parameters (serum creatinine, bilirubin, albumin).

†Pooled results calculated using Rubin's rules across 5 imputed datasets. OR, odds ratio; CI, confidence interval. All statistical analyses were performed using Python version 3.10.12.

Supplemental Table S6. Missing Data Analysis for Secondary Outcome (90-Day Mortality)

Variable	Complete Cases (n)	Missing Cases (n)	Missing Rate (%)	Handling Method
**Demographics and Comorbidities**				
Age	673	0	0.00	missForest
Sex	673	0	0.00	missForest
Diabetes mellitus	673	0	0.00	missForest
Hypertension	673	0	0.00	missForest
Chronic obstructive pulmonary disease	673	0	0.00	missForest
Congestive heart failure	673	0	0.00	missForest
Chronic kidney disease	673	0	0.00	missForest
Chronic liver disease	673	0	0.00	missForest
Cerebrovascular accident	673	0	0.00	missForest
**Anthropometric Measures**				
BMI	544	129	19.17	missForest
**Disease Severity**				
APACHE II score	598	75	11.14	missForest
**Biomarkers**				
Procalcitonin	620	53	7.88	missForest
C-reactive protein	673	0	0.00	missForest
**Vital Signs and Laboratory**				
Heart rate	673	0	0.00	missForest
Mean arterial pressure	672	1	0.15	missForest
Anion gap	673	0	0.00	missForest
Serum Creatinine	673	0	0.00	missForest
Blood urea nitrogen	673	0	0.00	missForest
**Organ Function**				
Lactate dehydrogenase	668	5	0.74	missForest

Variable	Complete Cases (n)	Missing Cases (n)	Missing Rate (%)	Handling Method
Albumin	669	4	0.59	missForest
Direct bilirubin	669	4	0.59	missForest
**Coagulation Parameters**				
Platelet count	672	1	0.15	missForest
International normalized ratio	672	1	0.15	missForest
**Acid-Base Status**				
Bicarbonate	672	1	0.15	missForest
**Treatment**				
Antibiotic therapy	673	0	0.00	missForest

Variables with missing rate ≥40% were excluded from analysis. Since multiple variables (BMI, APACHE II score, Procalcitonin) had missing rates >5%, all covariates were imputed using the missForest algorithm for methodological consistency, regardless of their individual missing rates. The missForest method was chosen for its ability to handle mixed-type data and capture complex variable interactions without distributional assumptions. Final sample size after processing: 673 patients. All statistical analyses were performed using Python version 3.10.12.

Supplemental Table S7. Comparison Between Original and Imputed Data for 90-Day Mortality Analysis

Variable	Original Data	Imp 1	Imp 2	Imp 3	Imp 4	Imp 5	Test	*p* value
**Disease Severity and Clinical Scores**								
APACHE II	22.60 ± 7.07 (n=598)	22.72 ± 7.23 (n=673)	22.47 ± 7.18 (n=673)	22.63 ± 7.09 (n=673)	22.36 ± 7.29 (n=673)	22.71 ± 7.03 (n=673)	ANOVA	0.931
**Anthropometric Measures**								
BMI	22.41 ± 4.05 (n=544)	22.27 ± 4.21 (n=673)	22.48 ± 4.21 (n=673)	22.44 ± 4.13 (n=673)	22.45 ± 4.15 (n=673)	22.45 ± 4.08 (n=673)	ANOVA	0.959
**Inflammatory Biomarkers**								
Procalcitonin	61.53 ± 67.31 (n=620)	61.25 ± 65.02 (n=673)	61.32 ± 65.03 (n=673)	61.23 ± 65.06 (n=673)	61.24 ± 65.02 (n=673)	61.31 ± 65.03 (n=673)	KW	0.997
C-reactive protein	144.99 ± 86.50 (n=673)	144.99 ± 86.50 (n=673)	144.99 ± 86.50 (n=673)	144.99 ± 86.50 (n=673)	144.99 ± 86.50 (n=673)	144.99 ± 86.50 (n=673)	N/A†	—
**Hepatic Function and Metabolism**								
Albumin	25.66 ± 6.48 (n=669)	25.62 ± 6.40 (n=673)	25.62 ± 6.39 (n=673)	25.61 ± 6.40 (n=673)	25.65 ± 6.38 (n=673)	25.65 ± 6.38 (n=673)	ANOVA	1.000
Direct bilirubin	18.98 ± 32.67 (n=669)	18.63 ± 30.79 (n=673)	18.77 ± 30.89 (n=673)	18.65 ± 30.79 (n=673)	18.64 ± 30.79 (n=673)	18.64 ± 30.79 (n=673)	KW	1.000
Lactate dehydrogenase	644.28 ± 1774.42 (n=668)	647.94 ± 1769.14 (n=673)	647.60 ± 1769.20 (n=673)	646.91 ± 1768.85 (n=673)	646.64 ± 1768.65 (n=673)	647.84 ± 1769.64 (n=673)	KW	1.000
**Acid-Base Status**								
Bicarbonate	19.30 ± 6.09 (n=672)	19.18 ± 5.21 (n=673)	19.20 ± 5.20 (n=673)	19.20 ± 5.20 (n=673)	19.20 ± 5.20 (n=673)	19.20 ± 5.21 (n=673)	ANOVA	1.000
Anion gap	15.27 ± 5.95 (n=673)	15.24 ± 5.75 (n=673)	15.24 ± 5.75 (n=673)	15.24 ± 5.75 (n=673)	15.24 ± 5.75 (n=673)	15.24 ± 5.75 (n=673)	N/A†	—
**Renal Function**								
Serum Creatinine	183.22 ± 166.45 (n=673)	183.22 ± 166.45 (n=673)	183.22 ± 166.45 (n=673)	183.22 ± 166.45 (n=673)	183.22 ± 166.45 (n=673)	183.22 ± 166.45 (n=673)	N/A†	—
Blood urea nitrogen	15.49 ± 22.15	15.49 ± 22.15	15.49 ± 22.15	15.49 ± 22.15	15.49 ± 22.15	15.49 ± 22.15	N/A†	—

Variable	Original Data	Imp 1	Imp 2	Imp 3	Imp 4	Imp 5	Test	*p* value
	(n=673)	(n=673)	(n=673)	(n=673)	(n=673)	(n=673)		
**Hemodynamic Parameters**								
Heart rate	108.16 ± 23.90 (n=673)	108.23 ± 23.19 (n=673)	108.23 ± 23.19 (n=673)	108.23 ± 23.19 (n=673)	108.23 ± 23.19 (n=673)	108.23 ± 23.19 (n=673)	N/A†	—
Mean arterial pressure	85.56 ± 19.12 (n=672)	85.54 ± 18.86 (n=673)	85.53 ± 18.86 (n=673)	85.52 ± 18.86 (n=673)	85.54 ± 18.86 (n=673)	85.51 ± 18.87 (n=673)	ANOVA	1.000
**Coagulation Parameters**								
Platelet count	162.77 ± 114.63 (n=672)	160.76 ± 104.87 (n=673)	160.88 ± 104.85 (n=673)	160.80 ± 104.85 (n=673)	160.78 ± 104.86 (n=673)	161.17 ± 105.20 (n=673)	ANOVA	1.000
International normalized ratio	1.49 ± 0.77 (n=672)	1.46 ± 0.48 (n=673)	1.46 ± 0.48 (n=673)	1.46 ± 0.48 (n=673)	1.46 ± 0.48 (n=673)	1.46 ± 0.48 (n=673)	ANOVA	1.000
**Demographics**								
Age	70.24 ± 14.37 (n=673)	70.25 ± 14.17 (n=673)	70.25 ± 14.17 (n=673)	70.25 ± 14.17 (n=673)	70.25 ± 14.17 (n=673)	70.25 ± 14.17 (n=673)	N/A†	—
**Sex**								
Male	422 (62.7%)	422 (62.7%)	422 (62.7%)	422 (62.7%)	422 (62.7%)	422 (62.7%)	N/A†	—
Female	251 (37.3%)	251 (37.3%)	251 (37.3%)	251 (37.3%)	251 (37.3%)	251 (37.3%)		
**Comorbidities**								
**Diabetes mellitus**								
No	438 (65.1%)	438 (65.1%)	438 (65.1%)	438 (65.1%)	438 (65.1%)	438 (65.1%)	N/A†	—
Yes	235 (34.9%)	235 (34.9%)	235 (34.9%)	235 (34.9%)	235 (34.9%)	235 (34.9%)		
**Hypertension**								
No	390 (57.9%)	390 (57.9%)	390 (57.9%)	390 (57.9%)	390 (57.9%)	390 (57.9%)	N/A†	—
Yes	283 (42.1%)	283 (42.1%)	283 (42.1%)	283 (42.1%)	283 (42.1%)	283 (42.1%)		
**Chronic obstructive pulmonary disease**								
No	630 (93.6%)	630 (93.6%)	630 (93.6%)	630 (93.6%)	630 (93.6%)	630 (93.6%)	N/A†	—
Yes	43 (6.4%)	43 (6.4%)	43 (6.4%)	43 (6.4%)	43 (6.4%)	43 (6.4%)		
**Congestive heart failure**								

Variable	Original Data	Imp 1	Imp 2	Imp 3	Imp 4	Imp 5	Test	*p* value
No	556 (82.6%)	556 (82.6%)	556 (82.6%)	556 (82.6%)	556 (82.6%)	556 (82.6%)	N/A†	—
Yes	117 (17.4%)	117 (17.4%)	117 (17.4%)	117 (17.4%)	117 (17.4%)	117 (17.4%)		
**Chronic kidney disease**								
No	481 (71.5%)	481 (71.5%)	481 (71.5%)	481 (71.5%)	481 (71.5%)	481 (71.5%)	N/A†	—
Yes	192 (28.5%)	192 (28.5%)	192 (28.5%)	192 (28.5%)	192 (28.5%)	192 (28.5%)		
**Chronic liver disease**								
No	666 (99.0%)	666 (99.0%)	666 (99.0%)	666 (99.0%)	666 (99.0%)	666 (99.0%)	N/A†	—
Yes	7 (1.0%)	7 (1.0%)	7 (1.0%)	7 (1.0%)	7 (1.0%)	7 (1.0%)		
**Cerebrovascular accident**								
No	572 (85.0%)	572 (85.0%)	572 (85.0%)	572 (85.0%)	572 (85.0%)	572 (85.0%)	N/A†	—
Yes	101 (15.0%)	101 (15.0%)	101 (15.0%)	101 (15.0%)	101 (15.0%)	101 (15.0%)		
**Treatment**								
**Antibiotic therapy**								
No antibacterial	19 (2.8%)	19 (2.8%)	19 (2.8%)	19 (2.8%)	19 (2.8%)	19 (2.8%)	N/A†	—
Narrow-spectrum	39 (5.8%)	39 (5.8%)	39 (5.8%)	39 (5.8%)	39 (5.8%)	39 (5.8%)		
Broad-spectrum	469 (69.7%)	469 (69.7%)	469 (69.7%)	469 (69.7%)	469 (69.7%)	469 (69.7%)		
Combination therapy	146 (21.7%)	146 (21.7%)	146 (21.7%)	146 (21.7%)	146 (21.7%)	146 (21.7%)		

Values for continuous variables are presented as mean ± standard deviation. Statistical comparisons were performed using one-way ANOVA for normally distributed variables or Kruskal-Wallis test (KW) for non-normally distributed variables. Categorical variables are presented as counts (percentage). †Not applicable for variables with no missing values. All analyses were performed using Python version 3.10.12 with the missForest algorithm for multiple imputation.

Supplemental Table S8. Multiple Imputation Analysis Results for Mechanical Ventilation and 90-Day Mortality

Analysis	Unadjusted OR (95% CI)	*p* value	Adjusted OR (95% CI)*	*p* value
Imputation 1	3.19 (2.19, 4.65)	<0.001	2.82 (1.81, 4.39)	<0.001
Imputation 2	3.19 (2.19, 4.65)	<0.001	2.86 (1.84, 4.45)	<0.001
Imputation 3	3.19 (2.19, 4.65)	<0.001	2.65 (1.70, 4.14)	<0.001
Imputation 4	3.19 (2.19, 4.65)	<0.001	2.77 (1.77, 4.33)	<0.001
Imputation 5	3.19 (2.19, 4.65)	<0.001	2.75 (1.76, 4.30)	<0.001
Pooled Result†	3.19 (2.19, 4.65)	<0.001	2.77 (1.77, 4.34)	<0.001

Results from each individual imputation dataset and pooled analysis using Rubin's rules.

*Adjusted for age, sex, APACHE II score, comorbidities (diabetes mellitus, hypertension, chronic obstructive pulmonary disease, congestive heart failure, chronic kidney disease, chronic liver disease, cerebrovascular accident), and laboratory parameters (serum creatinine, bilirubin, albumin).

†Pooled results calculated using Rubin's rules across 5 imputed datasets. OR, odds ratio; CI, confidence interval. All statistical analyses were performed using Python version 3.10.12.

Supplemental Table S9. Univariate Logistic Regression Analysis for 28-day and 90-day Mortality (OR with 3 Decimal Places)

Variable	Overall (n=673)	28-day Mortality OR (95% CI), *p* value	90-day Mortality OR (95% CI), *p* value
**Demographics**			
MV (Yes vs No)	507 (75.3%)	3.041 (2.09, 4.43), <0.001	3.191 (2.19, 4.65), <0.001
Age, years	70.25 ± 14.17	1.021 (1.01, 1.03), <0.001	1.023 (1.01, 1.04), <0.001
Sex (Female vs Male)	251 (37.3%)	1.021 (0.75, 1.40), 0.898	0.940 (0.69, 1.29), 0.698
BMI, kg/m²	22.30 ± 4.16	1.006 (0.96, 1.05), 0.766	1.009 (0.97, 1.05), 0.693
**Comorbidities**			
DM (Yes vs No)	235 (34.9%)	0.993 (0.72, 1.36), 0.964	1.100 (0.80, 1.51), 0.558
HTN (Yes vs No)	283 (42.1%)	1.075 (0.79, 1.46), 0.643	1.115 (0.82, 1.51), 0.487
COPD (Yes vs No)	43 (6.4%)	1.787 (0.94, 3.38), 0.074	1.720 (0.91, 3.26), 0.096
CHF (Yes vs No)	117 (17.4%)	1.401 (0.94, 2.09), 0.100	1.732 (1.15, 2.60), 0.008
CKD (Yes vs No)	192 (28.5%)	1.552 (1.11, 2.18), 0.011	1.714 (1.22, 2.41), 0.002
CLD (Yes vs No)	7 (1.0%)	2.576 (0.50, 13.37), 0.260	2.485 (0.48, 12.90), 0.279
CVA (Yes vs No)	101 (15.0%)	1.325 (0.87, 2.03), 0.194	1.332 (0.87, 2.04), 0.187
**Clinical Severity**			
APACHE II	22.40 ± 7.13	1.053 (1.03, 1.08), <0.001	1.054 (1.03, 1.08), <0.001
Heart rate, bpm	108.23 ± 23.19	1.014 (1.01, 1.02), <0.001	1.013 (1.01, 1.02), <0.001
MAP, mmHg	85.56 ± 18.86	0.994 (0.99, 1.00), 0.128	0.996 (0.99, 1.00), 0.350
**Laboratory Parameters**			
PLT, $\times 10^9$/L	145.00 (84.00-218.00)	0.997 (1.00, 1.00), <0.001	0.997 (1.00, 1.00), <0.001
INR	1.46 ± 0.48	2.202 (1.54, 3.14), <0.001	2.018 (1.42, 2.86), <0.001
Albumin, g/L	25.63 ± 6.37	0.974 (0.95, 1.00), 0.032	0.982 (0.96, 1.01), 0.137
DBIL, μmol/L	8.00 (4.60-17.00)	1.009 (1.00, 1.02), 0.002	1.007 (1.00, 1.01), 0.012
HCO_3^-, mmol/L	19.20 ± 5.20	0.980 (0.95, 1.01), 0.175	0.987 (0.96, 1.02), 0.389
AG, mmol/L	15.24 ± 5.75	1.075 (1.04, 1.11), <0.001	1.074 (1.04, 1.11), <0.001

Variable	Overall (n=673)	28-day Mortality OR (95% CI), *p* value	90-day Mortality OR (95% CI), *p* value
SCr, μmol/L	132.00 (86.00-217.00)	1.001 (1.00, 1.00), 0.028	1.002 (1.00, 1.00), 0.005
BUN, mmol/L	11.90 (7.70-18.60)	1.032 (1.02, 1.05), <0.001	1.034 (1.02, 1.05), <0.001
LDH, IU/L	277.00 (199.00-481.00)	1.000 (1.00, 1.00), <0.001	1.000 (1.00, 1.00), <0.001
PCT, ng/mL	38.39 (5.45-100.00)	0.999 (1.00, 1.00), 0.619	0.999 (1.00, 1.00), 0.411
CRP, mg/L	155.75 (71.86-200.00)	1.000 (1.00, 1.00), 0.641	0.999 (1.00, 1.00), 0.383
**Treatment (Antibiotic)**			
Narrow-spectrum vs None	39 (5.8%)	0.253 (0.07, 0.90), 0.034	0.311 (0.09, 1.11), 0.072
Broad-spectrum vs None	469 (69.7%)	0.234 (0.08, 0.71), 0.011	0.234 (0.08, 0.71), 0.011
Combination vs None	146 (21.7%)	0.323 (0.10, 1.02), 0.054	0.361 (0.11, 1.14), 0.083

Data are presented as mean ± standard deviation (SD) for normally distributed continuous variables, median [interquartile range, IQR] for non-normally distributed variables, and number (percentage) for categorical variables. Univariate logistic regression models were used to estimate odds ratios (OR) and 95% confidence intervals (CI) for 28-day and 90-day mortality. For categorical variables, the reference group was defined as the absence of the condition (e.g., "No") or the first category listed. A two-sided p value < 0.05 was considered statistically significant.

Abbreviations: OR, odds ratio; CI, confidence interval; MV, mechanical ventilation; BMI, body mass index; DM, diabetes mellitus; HTN, hypertension; COPD, chronic obstructive pulmonary disease; CHF, congestive heart failure; CKD, chronic kidney disease; CLD, chronic liver disease; APACHE II, Acute Physiology and Chronic Health Evaluation II; MAP, mean arterial pressure; PLT, platelets; INR, international normalized ratio; DBIL, direct bilirubin; AG, anion gap; SCr, serum creatinine; BUN, blood urea nitrogen; LDH, lactate dehydrogenase; PCT, procalcitonin; CRP, C-reactive protein.

Note: This table presents OR values with 3 decimal places as requested by the reviewer. For the main manuscript, OR values are reported with 2 decimal places per journal guidelines.
